# Clinical outcomes during the 2025-2026 and 2024-2025 influenza seasons in a US EHR network: an ecological comparison during A(H3N2) subclade K predominance

**DOI:** 10.1016/j.ijregi.2026.101025

**Published:** 2026-09-29

**Authors:** Taito Kitano, Sayaka Yoshida

**Affiliations:** 1 Department of Pediatrics, Nara Prefecture General Medical Center, Nara, Japan; 2 AMR Clinical Reference Center, Japan Institute for Health Security, Tokyo, Japan

**Keywords:** Influenza, Subclade K, H3N2, Electronic health records, Disease severity, Propensity score

## Abstract

**Objectives:**

The influenza A(H3N2) subclade K virus has predominated in the 2025-2026 season. This study compared the clinical severity of influenza between 2025-2026 and 2024-2025 seasons.

**Methods:**

In this retrospective cohort study, a multicenter electronic health record database (TriNetX) was used. The clinical outcomes after influenza infection confirmed by nucleic acid detection were ecologically compared between the 2025-2026 and 2024-2025 seasons with propensity score matching.

**Results:**

There were 8965 and 8937 children and 7488 and 6947 adults with confirmed influenza A(H3N2) infection in the 2025-2026 and 2024-2025 seasons, respectively. After each propensity score matching, the odds ratios for children and adults were not assessed (due to the small number of events) and 0.87 (95% confidence interval 0.71-1.05), *P* = 0.148 for death; 1.32 (1.10-1.59), *P* = 0.003 and 0.91 (0.82-1.02), *P* = 0.092 for critical care admission; 1.06 (0.97-1.16), *P* = 0.191 and 1.01 (0.94-1.08), *P* = 0.803 for hospitalization; 0.88 (0.73-1.07), *P* = 0.197 and 0.93 (0.83-1.04), *P* = 0.194 for mechanical ventilation; and 1.10 (0.91-1.32), *P* = 0.320 and 0.93 (0.83-1.03), *P* = 0.177 for vasopressor use, respectively.

**Conclusion:**

In this electronic health record–based season-level comparison, no consistent adjusted differences were observed; however, the study could not directly assess subclade K-specific severity or rule out clinically relevant differences. Rapid and timely monitoring of clinical characteristics and severity in the case of an emerging influenza strain or subclade is important to guide preventive and therapeutic strategies against influenza.

## Highlights

• In this electronic health record–based season-level comparison, no consistent adjusted differences were observed.
• The study could not directly assess subclade K-specific severity.
• Timely monitoring of clinical characteristics of an emerging strain is important.

## Background

Influenza continues to be an important public health threat. In July 2025, the influenza A(H3N2) subclade K virus was detected [1], and it has been the predominant strain in the 2025-2026 season in the northern hemisphere [2]. Although vaccination is a critical strategy to prevent severe outcomes associated with influenza, some data suggest a reduced vaccine effectiveness against the influenza A(H3N2) subclade K virus [3]. Given the new evolution acquired by the emerging subclade K virus, whether its clinical outcomes differ significantly from those of previous influenza seasons is an important question.

A recent study from Thailand with approximately 1100 patients with influenza showed that the risk of hospitalization in cases with influenza A(H3N2) subclade K virus was not statistically different from that of other influenza subclades compared with all other subclades or compared with other A(H3N2) subclades [4]. However, the risk of other outcomes (e.g. mortality, critical care admission, mechanical ventilation) could not be compared due to the small sample size in this study. Another early study from Australia and New Zealand about subclade K virus also could not compare the risk of critical outcomes [1].

Our study aimed to compare the risk of severe outcomes, including death, critical care admission, hospitalization, mechanical ventilation, and vasopressor use, between the 2025-2026 and 2024-2025 seasons using a multicenter database.

## Methods

In this retrospective cohort study, a multicenter electronic health record database (TriNetX) in the US network (137 million patients from 75 health care organizations) was used [5]. TriNetX is a health research platform that collects de-identified electronic health records from hundreds of health care organizations to accelerate clinical trials, perform real-world data studies, and analyze patient populations [5].

The eligible participants were patients with influenza A(H3N2) confirmed by nucleic acid detection between August 1, 2025 and April 30, 2026 for the 2025-2026 cohort and between August 1, 2024 and April 30, 2025 for the 2024-2025 cohort (Supplementary Table 1). Children (aged <18 years) and adults (≥18 years) were separately analyzed. Clinical characteristics and outcomes were assessed between the 2025-2026 and 2024-2025 cohorts. The primary outcome was death, and the secondary outcomes included critical care admission, hospitalization, mechanical ventilation, and vasopressor use (Supplementary Table 2). The clinical outcomes were followed up until 6 weeks since the confirmation of infection. Data were extracted on September 16, 2026. Institutional Review Board of Nara Prefecture General Medical Center reviewed and approved the study on July 14, 2026, with a waiver of written informed consent (No. 1111). The statistical analyses were described in Supplementary Methods and Supplemental Table 3 [[6], [7]].

## Results

There were 8965 (39 centers) and 8937 (39 centers) children and 7488 (39 centers) and 6947 (39 centers) adults with confirmed influenza A (H3N2) infection in the 2025-2026 and 2024-2025 seasons, respectively (Figure 1). Median ages were 4 and 4 years for the pediatric cohort and 59 and 57 years for the adult cohort in the 2025-2026 and 2024-2025 seasons, respectively. In the exploratory analyses, the numbers of patients with confirmed influenza A were 73,184 and 109,324 for children and 89,101 and 167,690 for adults, and those with confirmed all influenza were 118,862 and 131,336 for children and 112,628 and 185,531 for adults in the 2025-2026 and 2024-2025 seasons, respectively. Other participants’ characteristics are presented in Supplementary Tables 4-9.

**Figure 1:**
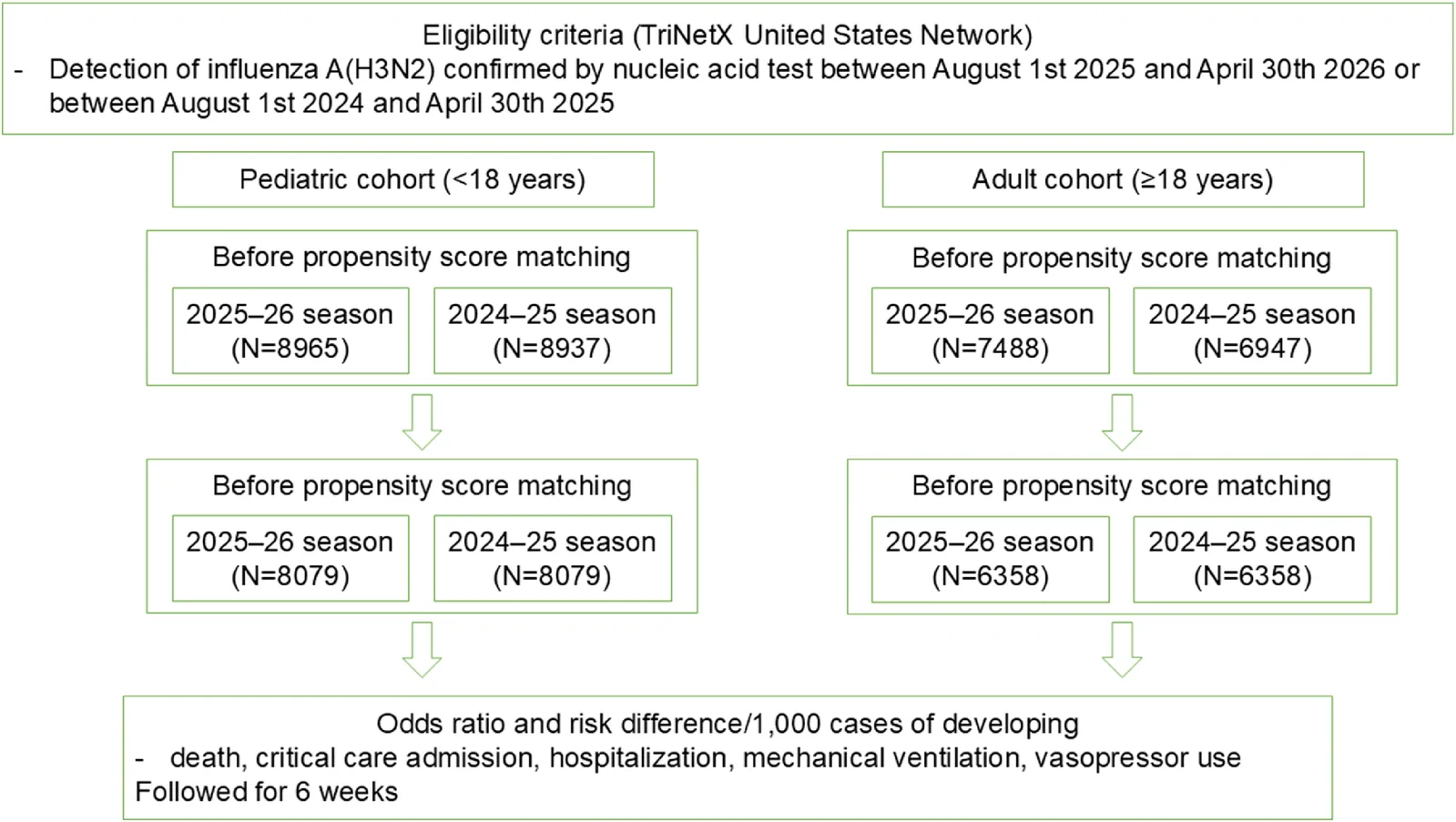
Participant flow for primary analysis, Influenza A(H3N2). All exploratory analyses were implemented with the same methodology.

After each propensity score matching, the standardized mean difference was <0.1 across all covariates in all analyses. The ORs and risk difference (RD) per 1000 cases in cases with confirmed influenza A(H3N2) were as follows, with odds ratio [OR] >1 or RD >0 indicating more outcome events in the 2025-2026 season (Table 1):

1. In children, OR was 1.32 (95% confidence interval 1.10-1.59), RD/1000 cases was 7.79 (2.36-14.30), and *P* = 0.003 for critical care admission; OR was 1.06 (0.97-1.16), RD/1000 cases was 7.21 (−3.47 to 18.72), and *P* = 0.191 for hospitalization; OR was 0.88 (0.73-1.07), RD/1000 cases was −3.34 (−7.72 to 1.89), and *P* = 0.197 for mechanical ventilation; and OR was 1.10 (0.91-1.32), RD/1000 cases was 2.60 (−2.30 to 8.48), and *P* = 0.320 for vasopressor use. The OR or RD for death could not be evaluated due to its small number of outcome events (≤10) in either group.
2. In adults, OR 0.87 (0.71-1.05), RD/1000 cases −4.57 (−9.81 to 1.78), and *P* = 0.148 for death; OR 0.91 (0.82-1.02), RD/1000 cases −9.92 (−20.50 to 1.70), *P* = 0.092 for critical care admission; OR 1.01 (0.94-1.08), RD/1000 cases 2.30 (−14.75 to 19.82), *P* = 0.803 for hospitalization; OR 0.93 (0.83-1.04), RD/1000 cases −7.23 (−17.34 to 3.90), *P* = 0.194 for mechanical ventilation; and OR 0.93 (0.83-1.03), RD/1000 cases −7.65 (−18.09 to 3.64), *P* = 0.177 for vasopressor use.

**Table 1:** Clinical outcomes of influenza A(H3N2) between the 2025-2026 and 2024-2025 seasons.

Outcome	Pediatric cohort				
	2025-2026 season (N = 8079)	2024-2025 season (N = 8079)	OR [95% CI]	RD/1000 cases [95% CI]	*P*
Death	NA	NA	NA	NA	NA
Critical care admission	266 (3.29%)	203 (2.51%)	1.32 [1.10-1.59]	7.79 [2.36-14.30]	0.003
Hospitalization	1178 (14.58%)	1120 (13.86%)	1.06 [0.97-1.16]	7.21 [−3.47 to 18.72]	0.191
Mechanical ventilation	212 (2.62%)	239 (2.96%)	0.88 [0.73-1.07]	−3.34 [−7.72 to 1.89]	0.197
Vasopressor	240 (2.97%)	219 (2.71%)	1.10 [0.91-1.32]	2.60 [−2.30 to 8.48]	0.320

	Adult cohort				
	2025-2026 season (N = 6358)	2024-2025 season (N = 6358)	OR [95% CI]	RD/1000 cases [95% CI]	*P*
Death	193 (3.04%)	222 (3.49%)	0.87 [0.71-1.05]	−4.57 [−9.81 to 1.78]	0.148
Critical care admission	767 (12.06%)	830 (13.05%)	0.91 [0.82-1.02]	−9.92 [−20.50 to 1.70]	0.092
Hospitalization	2944 (46.30%)	2930 (46.08%)	1.01 [0.94-1.08]	2.30 [−14.75 to 19.82]	0.803
Mechanical ventilation	684 (10.76%)	730 (11.48%)	0.93 [0.83-1.04]	−7.23 [−17.34 to 3.90]	0.194
Vasopressor	723 (11.37%)	772 (12.14%)	0.93 [0.83-1.03]	−7.65 [−18.09 to 3.64]	0.177

Abbreviations: CI, confidence interval; NA, not assessed; OR, odds ratio; RD, risk difference.

The findings of all exploratory analyses are presented in Supplementary Tables 10-19.

## Discussion

The study evaluated the clinical outcomes of influenza in the 2025-2026 season, when the subclade K predominated, compared with those of the 2024-2025 season. No consistent trend of statistical significance in the ORs was observed across the five investigated clinical severity outcomes. The findings in this study were similar to those of relevant studies that could only compare the more common severe outcomes (i.e. hospitalization), given that their findings were in an early phase with relatively small sample sizes [1,4].

Some studies suggested that the effectiveness of vaccines against influenza A (H3N2) subclade K could be maintained [8]. However, our study could not optimally identify vaccinated patients. In this study, the impact of antigenic mismatch, immunologic cross-protection, and clinical vaccine effectiveness on the observed severity data could not be evaluated. The implications of this study include the importance of timely assessments of clinical characteristics and severity in the case of an emerging strain.

The study was limited by some important points. Because the study ecologically compared clinical outcomes between the two influenza seasons without access to sequencing data, it did not directly estimate the virulence or severity of subclade K infection. Although the study performed propensity score matchings with a number of covariates, including participants’ demographics and comorbidities, residual confounding factors could have impacted the findings. Regarding comorbidities, “unknown” and “no comorbidity” could not be disaggregated in the database. The study could not account for a number of important factors, including difference in timing and intensity of epidemics, pathogens coinfected with influenza, previous population immunity, vaccine composition and uptake, influenza testing behavior and practice, antiviral use, admission thresholds, health care capacity and utilization, geographical variability, and coding practices. Thus, whether the observed findings were directly due to subclade K could not be concluded. Because some centers performed influenza tests without identifying the subtype, H3N2 subtype data were available for only a small fraction of participants. Therefore, the power to detect a difference in clinical outcomes between the two seasons for the influenza A (H3N2) was not high. Because the study used data of electronic health records mostly from large academic organizations, the study findings were less likely to represent the status of small facilities and clinics in which mildly symptomatic patients or low-risk patients could account for a larger proportion compared with large facilities.

In summary, in this electronic health record–based season-level comparison, no consistent adjusted differences were observed; however, the study could not directly assess subclade K-specific severity or rule out clinically relevant differences. Rapid and timely monitoring of clinical characteristics and severity in the case of an emerging influenza strain or subclade is important to guide preventive and therapeutic strategies against influenza.

## Funding

This research did not receive any specific grant from funding agencies in the public, commercial, or not-for-profit sectors.

## Ethical approval

Institutional review board at Nara Prefecture General Medical Center approved the study’s publication with a waiver of written informed consent.

## Data availability

Although the aggregate query definitions, code lists, and analytical syntax are presented in the supplementary tables, the full file can be shared upon reasonable request to corresponding author. Individual-level data are not available because of the regulation for participant’s privacy protection.

## Consent for publication

Not applicable.

## CRediT authorship contribution statement

**Taito Kitano:** Writing – original draft, Visualization, Validation, Software, Resources, Project administration, Methodology, Investigation, Formal analysis, Data curation, Conceptualization. **Sayaka Yoshida:** Writing – review & editing, Visualization, Validation, Supervision, Methodology, Investigation, Conceptualization.

## Declaration of competing interest

Dr Kitano received grants from bioMérieux. Dr Yoshida received grants from Tauns Laboratories and Mizuho Medy.

## Appendix Supplementary materials

Image, application 1
