# Supplementary material for: Clinical outcomes during the 2025-2026 and 2024-2025 influenza seasons in a US EHR network: an ecological comparison during A(H3N2) subclade K predominance
Source: IJID Reg. 2026 Sep 29;21:101025. doi: 10.1016/j.ijregi.2026.101025 (PMC13693347; doi:10.1016/j.ijregi.2026.101025)
Supplement: Supplementary file 1 [file mmc1.docx]

**Supplemental Methods**

*Statistical analysis*

For each patient, only first infection in each season was included. Propensity score matching was performed to compare each clinical outcome. Propensity score was estimated a standard unconditional logistic regression model, and greedy nearest-neighbor matching without replacement was implemented with 1:1 ratio, order randomization, and 0.1 pooled standard deviations. Age group, sex, race, ethnicity and presence of each designated comorbidity (only diagnosis before the date of positive result) were matched in each analysis. No missing data was observed for age, whereas unknown race and ethnicity variables were included in the matching. Respective comorbidity covariates (extracted based on ICD-10) included in each matching were selected from relevant pediatric and adult studies (Supplemental Table 3) [6,7].

OR of developing each clinical outcome was assessed from 2x2 contingency table with OR>1 indicating more severe outcome in the 2025–2026 season, while matched-pair correlation was not accounted for. p=0.005 was set as the cutoff for statistical significance to avoid type 1 error following the Bonferroni correction (0.05/510.

The following exploratory analyses were implemented. Subgroup analyses were performed according to the documented vaccination for the season (i.e., between 14 days and 8 months before infection), the receipt of any anti-influenza medications (e.g., oseltamivir, zanamivir, laninamivir, peramivir, and baloxavir), and type of healthcare organization (i.e., academic and non-academic). A sensitivity analysis was performed by excluding cases in which outcome events occurred on the same day as a positive result.

Supplemental Table 1. Cohort extraction code for all influenza confirmed by nucleic acid testing in children in the 2025–2026 season.

| Definition | | | | |
| --- | --- | --- | --- | --- |
| must have | any of | laboratory | UMLS:LNC:82168-6 | Influenza virus A H1 2009 pandemic RNA [Presence] in Nasopharynx by NAA with non-probe detection (at most 17 years old at event; labResult: Positive) |
|  |  | laboratory | UMLS:LNC:34487-9 | Influenza virus A RNA [Presence] in Specimen by NAA with probe detection (at most 17 years old at event; labResult: Positive) |
|  |  | laboratory | UMLS:LNC:92142-9 | Influenza virus A RNA [Presence] in Respiratory system specimen by NAA with probe detection (at most 17 years old at event; labResult: Positive) |
|  |  | laboratory | UMLS:LNC:92141-1 | Influenza virus B RNA [Presence] in Respiratory system specimen by NAA with probe detection (at most 17 years old at event; labResult: Positive) |
|  |  | laboratory | UMLS:LNC:76080-1 | Influenza virus B RNA [Presence] in Nasopharynx by NAA with probe detection (at most 17 years old at event; labResult: Positive) |
|  |  | laboratory | UMLS:LNC:40982-1 | Influenza virus B RNA [Presence] in Specimen by NAA with probe detection (at most 17 years old at event; labResult: Positive) |
|  |  | laboratory | UMLS:LNC:76078-5 | Influenza virus A RNA [Presence] in Nasopharynx by NAA with probe detection (at most 17 years old at event; labResult: Positive) |
|  |  | laboratory | UMLS:LNC:82170-2 | Influenza virus B RNA [Presence] in Nasopharynx by NAA with non-probe detection (at most 17 years old at event; labResult: Positive) |
|  |  | laboratory | UMLS:LNC:82166-0 | Influenza virus A RNA [Presence] in Nasopharynx by NAA with non-probe detection (at most 17 years old at event; labResult: Positive) |
|  |  | laboratory | UMLS:LNC:49524-2 | Influenza virus A H3 RNA [Presence] in Specimen by NAA with probe detection (at most 17 years old at event; labResult: Positive) |
|  |  | laboratory | UMLS:LNC:49521-8 | Influenza virus A H1 RNA [Presence] in Specimen by NAA with probe detection (at most 17 years old at event; labResult: Positive) |
|  |  | laboratory | UMLS:LNC:62462-7 | Influenza virus A+B RNA [Presence] in Specimen by NAA with probe detection (at most 17 years old at event; labResult: Positive) |
|  |  | laboratory | UMLS:LNC:85478-6 | Influenza virus B RNA [Presence] in Upper respiratory specimen by NAA with probe detection (at most 17 years old at event; labResult: Positive) |
|  |  | laboratory | UMLS:LNC:55465-9 | Influenza virus A H1 2009 pandemic RNA [Presence] in Specimen by NAA with probe detection (at most 17 years old at event; labResult: Positive) |
|  |  | laboratory | UMLS:LNC:85477-8 | Influenza virus A RNA [Presence] in Upper respiratory specimen by NAA with probe detection (at most 17 years old at event; labResult: Positive) |
|  |  | laboratory | UMLS:LNC:38381-0 | Influenza virus A cDNA [Presence] in Specimen by NAA with probe detection (at most 17 years old at event; labResult: Positive) |
|  |  | laboratory | UMLS:LNC:77026-3 | Influenza virus A H1 RNA [Presence] in Nasopharynx by NAA with probe detection (at most 17 years old at event; labResult: Positive) |
|  |  | laboratory | UMLS:LNC:82169-4 | Influenza virus A H3 RNA [Presence] in Nasopharynx by NAA with non-probe detection (at most 17 years old at event; labResult: Positive) |
|  |  | laboratory | UMLS:LNC:77028-9 | Influenza virus A H1 2009 pandemic RNA [Presence] in Nasopharynx by NAA with probe detection (at most 17 years old at event; labResult: Positive) |
|  |  | laboratory | UMLS:LNC:77027-1 | Influenza virus A H3 RNA [Presence] in Nasopharynx by NAA with probe detection (at most 17 years old at event; labResult: Positive) |
|  |  | laboratory | UMLS:LNC:82167-8 | Influenza virus A H1 RNA [Presence] in Nasopharynx by NAA with non-probe detection (at most 17 years old at event; labResult: Positive) |
|  |  | laboratory | UMLS:LNC:94395-1 | Influenza virus A H1 2009 pandemic RNA [Presence] in Lower respiratory specimen by NAA with probe detection (at most 17 years old at event; labResult: Positive) |
|  |  | laboratory | UMLS:LNC:76079-3 | Influenza virus B RNA [Presence] in Bronchoalveolar lavage by NAA with probe detection (at most 17 years old at event; labResult: Positive) |
|  |  | laboratory | UMLS:LNC:92976-0 | Influenza virus B RNA [Presence] in Lower respiratory specimen by NAA with non-probe detection (at most 17 years old at event; labResult: Positive) |
|  |  | laboratory | UMLS:LNC:48310-7 | Influenza virus A [Presence] in Specimen by Organism specific culture (at most 17 years old at event; labResult: Positive) |
|  |  | laboratory | UMLS:LNC:92977-8 | Influenza virus A RNA [Presence] in Lower respiratory specimen by NAA with non-probe detection (at most 17 years old at event; labResult: Positive) |
|  |  | laboratory | UMLS:LNC:80588-7 | Influenza virus A M gene [Presence] in Nasopharynx by NAA with probe detection (at most 17 years old at event; labResult: Positive) |
|  |  | laboratory | UMLS:LNC:49520-0 | Influenza virus A H1 RNA [Presence] in Isolate by NAA with probe detection (at most 17 years old at event; labResult: Positive) |
|  |  | laboratory | UMLS:LNC:92809-3 | Influenza virus A H1 RNA [Presence] in Upper respiratory specimen by NAA with probe detection (at most 17 years old at event; labResult: Positive) |
|  |  | laboratory | UMLS:LNC:80589-5 | Influenza virus A H1 HA gene [Presence] in Nasopharynx by NAA with probe detection (at most 17 years old at event; labResult: Positive) |
|  |  | laboratory | UMLS:LNC:76077-7 | Influenza virus A RNA [Presence] in Bronchoalveolar lavage by NAA with probe detection (at most 17 years old at event; labResult: Positive) |
|  |  | laboratory | UMLS:LNC:94396-9 | Influenza virus A H1 RNA [Presence] in Lower respiratory specimen by NAA with probe detection (at most 17 years old at event; labResult: Positive) |
|  |  | laboratory | UMLS:LNC:55464-2 | Influenza virus A swine origin RNA [Presence] in Specimen by NAA with probe detection (at most 17 years old at event; labResult: Positive) |
|  |  | laboratory | UMLS:LNC:60538-6 | Influenza virus A H1+H3+B RNA [Presence] in Specimen by NAA with probe detection (at most 17 years old at event; labResult: Positive) |
|  |  | laboratory | UMLS:LNC:88592-1 | Influenza virus B RNA [Presence] in Lower respiratory specimen by NAA with probe detection (at most 17 years old at event; labResult: Positive) |
|  |  | laboratory | UMLS:LNC:94394-4 | Influenza virus A H3 RNA [Presence] in Lower respiratory specimen by NAA with probe detection (at most 17 years old at event; labResult: Positive) |
|  |  | laboratory | UMLS:LNC:49535-8 | Influenza virus B RNA [Presence] in Isolate by NAA with probe detection (at most 17 years old at event; labResult: Positive) |
|  |  | laboratory | UMLS:LNC:49531-7 | Influenza virus A RNA [Presence] in Isolate by NAA with probe detection (at most 17 years old at event; labResult: Positive) |
|  |  | laboratory | UMLS:LNC:38382-8 | Influenza virus B [Presence] in Specimen by Organism specific culture (at most 17 years old at event; labResult: Positive) |
|  |  | laboratory | UMLS:LNC:80590-3 | Influenza virus A H3 HA gene [Presence] in Nasopharynx by NAA with probe detection (at most 17 years old at event; labResult: Positive) |
|  |  | laboratory | UMLS:LNC:92808-5 | Influenza virus A H3 RNA [Presence] in Upper respiratory specimen by NAA with probe detection (at most 17 years old at event; labResult: Positive) |
|  |  | laboratory | UMLS:LNC:49523-4 | Influenza virus A H3 RNA [Presence] in Isolate by NAA with probe detection (at most 17 years old at event; labResult: Positive) |
| date constraint | | The terms in this group occurred between Aug 1, 2025 and Apr 30, 2026 | | |

Other cohorts were also developed by amending each corresponding section.

Supplemental Table 2. Definition of outcomes

| Death | | | |
| --- | --- | --- | --- |
|  |  | Demographics | Deceased |
| Critical care admission | | | |
|  | Procedure | UMLS:CPT:1013729 | Critical Care Services |
|  | Procedure | UMLS:CPT:1019130 | Inpatient Neonatal Intensive Care Services and Pediatric and Neonatal Critical Care Services |
|  | Procedure | UMLS:CPT:1019134 | Inpatient Neonatal and Pediatric Critical Care |
|  | Procedure | UMLS:SNOMED:305351004 | Admission to intensive care unit |
|  | Procedure | UMLS:SNOMED:830077005 | Admission to neonatal intensive care unit |
|  | Procedure | UMLS:CPT:1019140 | Initial and Continuing Intensive Care Services |
| Hospitalization | | | |
|  | Visit | UMLS:HL7V3.0:VisitType:IMP | Visit: Inpatient Encounter |
|  | Visit | UMLS:HL7V3.0:VisitType:NONAC | Visit: Inpatient Non-acute |
|  | Visit | UMLS:HL7V3.0:VisitType:SS | Visit: Short Stay |
|  | Procedure | UMLS:CPT:1013659 | Hospital Inpatient and Observation Care Services |
|  | Procedure | UMLS:CPT:1013660 | Initial Hospital Inpatient or Observation Care |
| Mechanical ventilation | | | |
|  | Procedure | UMLS:ICD10PCS:5A09357 | Assistance with Respiratory Ventilation, Less than 24 Consecutive Hours, Continuous Positive Airway Pressure |
|  | Procedure | UMLS:ICD10PCS:5A09457 | Assistance with Respiratory Ventilation, 24-96 Consecutive Hours, Continuous Positive Airway Pressure |
|  | Procedure | UMLS:ICD10PCS:5A09557 | Assistance with Respiratory Ventilation, Greater than 96 Consecutive Hours, Continuous Positive Airway Pressure |
|  | Procedure | UMLS:ICD10PCS:5A1945Z | Respiratory Ventilation, 24-96 Consecutive Hours |
|  | Procedure | UMLS:ICD10PCS:5A1935Z | Respiratory Ventilation, Less than 24 Consecutive Hours |
|  | Procedure | UMLS:ICD10PCS:5A1955Z | Respiratory Ventilation, Greater than 96 Consecutive Hours |
|  | Procedure | UMLS:SNOMED:40617009 | Artificial ventilation |
|  | Procedure | UMLS:ICD10PCS:5A0935Z | Assistance with Respiratory Ventilation, Less than 24 Consecutive Hours |
|  | Procedure | UMLS:ICD10PCS:5A0945Z | Assistance with Respiratory Ventilation, 24-96 Consecutive Hours |
|  | Procedure | UMLS:ICD10PCS:5A0955Z | Assistance with Respiratory Ventilation, Greater than 96 Consecutive Hours |
|  | Procedure | UMLS:ICD10PCS:5A09459 | Assistance with Respiratory Ventilation, 24-96 Consecutive Hours, Continuous Negative Airway Pressure |
|  | Procedure | UMLS:ICD10PCS:5A09359 | Assistance with Respiratory Ventilation, Less than 24 Consecutive Hours, Continuous Negative Airway Pressure |
|  | Procedure | UMLS:ICD10PCS:5A0945B | Assistance with Respiratory Ventilation, 24-96 Consecutive Hours, Intermittent Negative Airway Pressure |
|  | Procedure | UMLS:ICD10PCS:5A09559 | Assistance with Respiratory Ventilation, Greater than 96 Consecutive Hours, Continuous Negative Airway Pressure |
|  | Procedure | UMLS:SNOMED:52765003 | Intubation |
|  | Procedure | UMLS:CPT:31500 | Intubation, endotracheal, emergency procedure |
|  | Procedure | UMLS:ICD10PCS:5A0945A | Assistance with Respiratory Ventilation, 24-96 Consecutive Hours, High Flow/Velocity Cannula |
|  | Procedure | UMLS:ICD10PCS:5A0935A | Assistance with Respiratory Ventilation, Less than 24 Consecutive Hours, High Flow/Velocity Cannula |
|  | Procedure | UMLS:ICD10PCS:5A0955A | Assistance with Respiratory Ventilation, Greater than 96 Consecutive Hours, High Flow/Velocity Cannula |
|  | Laboratory | UMLS:LNC:52042-9 | Continuous positive airway pressure (CPAP) attachment |
|  | Procedure | UMLS:CPT:94660 | Continuous positive airway pressure ventilation (CPAP), initiation and management |
|  | Laboratory | UMLS:LNC:58959-8 | BIPAP AndOr CPAP setting Ventilator ( (most recent occurrence)) |
| Pressor use | | | |
|  | Medication | NLM:VA:AU100 | SYMPATHOMIMETICS (ADRENERGICS) |
|  | Procedure | UMLS:ICD10PCS:3E033XZ | Introduction of Vasopressor into Peripheral Vein, Percutaneous Approach |
|  | Procedure | UMLS:ICD10PCS:3E043XZ | Introduction of Vasopressor into Central Vein, Percutaneous Approach |
|  | Procedure | UMLS:ICD10PCS:3E053XZ | Introduction of Vasopressor into Peripheral Artery, Percutaneous Approach |
|  | Procedure | UMLS:ICD10PCS:3E030XZ | Introduction of Vasopressor into Peripheral Vein, Open Approach |
|  | Procedure | UMLS:ICD10PCS:3E063XZ | Introduction of Vasopressor into Central Artery, Percutaneous Approach |
|  | Procedure | UMLS:ICD10PCS:3E040XZ | Introduction of Vasopressor into Central Vein, Open Approach |
|  | Procedure | UMLS:ICD10PCS:3E060XZ | Introduction of Vasopressor into Central Artery, Open Approach |
|  | Procedure | UMLS:ICD10PCS:3E050XZ | Introduction of Vasopressor into Peripheral Artery, Open Approach |

Supplemental Table 3. Definition of covariates included in propensity score matching

| Covariates | Coding definition |
| --- | --- |
| High-risk socioeconomic and psychosocial circumstances | ICD-10 Z55-Z65 |
| Long term (current) use of steroids | ICD-10 Z79.5 |
| Long term (current) use of immunomodulators and immunosuppressants | ICD-10 Z79.6 |
| Long term (current) use of non-steroidal anti-inflammatories (NSAID) | ICD-10 Z79.1 |
| Transplanted organ and tissue status | ICD-10 Z94 |
| Chronic lower respiratory diseases | ICD-10 J40-J4A |
| Tuberculosis | ICD-10 A15-A19 |
| Human immunodeficiency virus infection | ICD-10 B20-B20 |
| Diseases of liver | ICD-10 K70-K77 |
| Developmental disorders | ICD-10 F80-F89 |
| Cystic fibrosis | ICD-10 E84 |
| Obesity | ICD-10 E66 |
| Diabetes mellitus | ICD-10 E08-E13 |
| Acute kidney failure and chronic kidney disease | ICD-10 N17-N19 |
| Congenital malformations of the circulatory system ^a^ | ICD-10 Q20-Q28 |
| Congenital malformations of the respiratory system ^a^ | ICD-10 Q30-Q34 |
| Congenital malformations of the nervous system ^a^ | ICD-10 Q00-Q07 |
| Other congenital malformations of the digestive system ^a^ | ICD-10 Q38-Q45 |
| Cerebral palsy and other paralytic syndromes | ICD-10 G80-G83 |
| Epilepsy and recurrent seizures | ICD-10 G40 |
| Diseases of myoneural junction and muscle | ICD-10 G70-G73 |
| Immunodeficiency and associated syndrome | ICD-10 D80-D89 |
| Sickle-cell disorders | ICD-10 D57 |
| Thalassemia | ICD-10 D56 |
| Preterm or premature birth ^a^ | ICD-10 P07.3 |
| Chronic respiratory disease originating in the perinatal period ^a^ | ICD-10 P27 |
| Heart failure | ICD-10 I50 |
| Inflammatory polyarthropathies ^b^ | ICD-10 M05-M14 |
| Systemic connective tissue disorders ^b^ | ICD-10 M30-M36 |
| Psoriasis ^b^ | ICD-10 L40 |
| Multiple sclerosis ^b^ | ICD-10 G35 |
| Parkinson's disease ^b^ | ICD-10 G20 |
| Ischemic heart diseases ^b^ | ICD-10 I20-I25 |
| Cerebrovascular diseases ^b^ | ICD-10 I60-I69 |
| Ulcerative colitis ^b^ | ICD-10 K51 |
| Crohn's disease ^b^ | ICD-10 K50 |
| Mood or affective disorders ^b^ | ICD-10 F30-F39 |
| Schizophrenia ^b^ | ICD-10 F20-F29 |
| Nicotine dependence ^b^ | ICD-10 F17 |
| Alcohol related disorders ^b^ | ICD-10 F10 |
| Opioid related disorders ^b^ | ICD-10 F11 |
| Antineoplastic use | VA AN000 |

Abbreviations: ICD-10, International Statistical Classification of Diseases and Related Health Problems 10th Revision.

^a^ Only for pediatric cohort

^b^ Only for adult cohort

Supplemental Table 4. Characteristics of influenza A(H3N2) confirmed by nucleic acid testing in children.

|  | Before matching | | | After matching | | |
| --- | --- | --- | --- | --- | --- | --- |
|  | 2025–2026 season (N=8965) | 2024–2025 season (N=8937) | SMD | 2025–2026 season (N=8079) | 2024–2025 season (N=8079) | SMD |
| Age, years |  |  |  |  |  |  |
| Median [IQR] | 4 (8) | 4 (6) | 0.019 | 4 (7) | 4 (7) | 0.013 |
| <1 | 1330 (14.8%) | 1093 (12.2%) |  | 1079 (13.4%) | 1072 (13.3%) |  |
| 1 year | 1070 (11.9%) | 1002 (11.2%) |  | 990 (12.3%) | 958 (11.9%) |  |
| 2–4 | 2291 (25.6%) | 2391 (26.8%) |  | 2148 (26.6%) | 2172 (26.9%) |  |
| 5–11 | 2930 (32.7%) | 3354 (37.5%) |  | 2782 (34.4%) | 2798 (34.6%) |  |
| 12–17 | 1344 (15.0%) | 1097 (12.3%) |  | 1080 (13.4%) | 1079 (13.4%) |  |
| Sex (Female) | 4156 (46.4%) | 4207 (47.1%) | 0.014 | 3770 (46.7%) | 3785 (46.8%) | 0.004 |
| Race |  |  |  |  |  |  |
| American Indian or Alaska Native | 80 (0.9%) | 50 (0.6%) | 0.039 | 47 (0.6%) | 50 (0.6%) | 0.005 |
| Asian | 274 (3.1%) | 281 (3.1%) | 0.005 | 262 (3.2%) | 252 (3.1%) | 0.007 |
| Black or African American | 2071 (23.1%) | 1907 (21.3%) | 0.042 | 1839 (22.8%) | 1825 (22.6%) | 0.004 |
| Native Hawaiian or Other Pacific Islander | 83 (0.9%) | 127 (1.4%) | 0.046 | 80 (1.0%) | 71 (0.9%) | 0.012 |
| White | 4161 (46.4%) | 4389 (49.1%) | 0.054 | 3904 (48.3%) | 3916 (48.5%) | 0.003 |
| Other Race | 938 (10.5%) | 1090 (12.2%) | 0.055 | 906 (11.2%) | 905 (11.2%) | <0.001 |
| Unknown Race | 1358 (15.1%) | 1093 (12.2%) | 0.085 | 1041 (12.9%) | 1060 (13.1%) | 0.007 |
| Ethnicity |  |  |  |  |  |  |
| Hispanic or Latino | 2668 (29.8%) | 2740 (30.7%) | 0.020 | 2369 (29.3%) | 2429 (30.1%) | 0.016 |
| Not Hispanic or Latino | 5686 (63.4%) | 5487 (61.4%) | 0.042 | 5114 (63.3%) | 5069 (62.7%) | 0.012 |
| Unknown Ethnicity | 611 (6.8%) | 710 (7.9%) | 0.043 | 596 (7.4%) | 581 (7.2%) | 0.007 |
| Medical comorbidities |  |  |  |  |  |  |
| High-risk socioeconomic and psychosocial circumstances | 736 (8.2%) | 589 (6.6%) | 0.062 | 572 (7.1%) | 570 (7.1%) | 0.001 |
| Long term (current) use of steroids | 455 (5.1%) | 393 (4.4%) | 0.032 | 360 (4.5%) | 367 (4.5%) | 0.004 |
| Long term (current) use of immunomodulators and immunosuppressants | 241 (2.7%) | 132 (1.5%) | 0.085 | 128 (1.6%) | 130 (1.6%) | 0.002 |
| Long term (current) use of non-steroidal anti-inflammatories | 86 (1.0%) | 82 (0.9%) | 0.004 | 72 (0.9%) | 71 (0.9%) | 0.001 |
| Transplanted organ and tissue status | 81 (0.9%) | 64 (0.7%) | 0.021 | 54 (0.7%) | 59 (0.7%) | 0.007 |
| Chronic lower respiratory diseases | 1585 (17.7%) | 1630 (18.2%) | 0.015 | 1428 (17.7%) | 1458 (18.0%) | 0.010 |
| Tuberculosis | 10 (0.1%) | 10 (0.1%) | <0.001 | 10 (0.1%) | 10 (0.1%) | <0.001 |
| Human immunodeficiency virus infection | 10 (0.1%) | 0 (0.0%) | 0.047 | 0 (0.0%) | 0 (0.0%) | -- |
| Diseases of liver | 142 (1.6%) | 110 (1.2%) | 0.030 | 104 (1.3%) | 103 (1.3%) | 0.001 |
| Pervasive and specific developmental disorders | 1428 (15.9%) | 1357 (15.2%) | 0.021 | 1224 (15.2%) | 1246 (15.4%) | 0.008 |
| Cystic fibrosis | 15 (0.2%) | 18 (0.2%) | 0.008 | 14 (0.2%) | 17 (0.2%) | 0.008 |
| Overweight and obesity | 433 (4.8%) | 390 (4.4%) | 0.022 | 358 (4.4%) | 369 (4.6%) | 0.007 |
| Diabetes mellitus | 86 (1.0%) | 66 (0.7%) | 0.024 | 68 (0.8%) | 62 (0.8%) | 0.008 |
| Acute kidney failure and chronic kidney disease | 251 (2.8%) | 201 (2.2%) | 0.035 | 182 (2.3%) | 185 (2.3%) | 0.002 |
| Congenital malformations of the circulatory system | 572 (6.4%) | 579 (6.5%) | 0.004 | 502 (6.2%) | 514 (6.4%) | 0.006 |
| Congenital malformations of the respiratory system | 253 (2.8%) | 232 (2.6%) | 0.014 | 209 (2.6%) | 214 (2.6%) | 0.004 |
| Congenital malformations of the nervous system | 235 (2.6%) | 185 (2.1%) | 0.036 | 174 (2.2%) | 178 (2.2%) | 0.003 |
| Congenital malformations of the digestive system | 279 (3.1%) | 243 (2.7%) | 0.023 | 227 (2.8%) | 230 (2.8%) | 0.002 |
| Cerebral palsy and other paralytic syndromes | 164 (1.8%) | 146 (1.6%) | 0.015 | 121 (1.5%) | 137 (1.7%) | 0.016 |
| Epilepsy and recurrent seizures | 406 (4.5%) | 299 (3.3%) | 0.061 | 281 (3.5%) | 295 (3.7%) | 0.009 |
| Diseases of myoneural junction and muscle | 59 (0.7%) | 43 (0.5%) | 0.024 | 41 (0.5%) | 42 (0.5%) | 0.002 |
| Immunodeficiency and associated syndromes | 356 (4.0%) | 267 (3.0%) | 0.054 | 249 (3.1%) | 257 (3.2%) | 0.006 |
| Sickle-cell disorders | 207 (2.3%) | 169 (1.9%) | 0.029 | 157 (1.9%) | 162 (2.0%) | 0.004 |
| Thalassemia | 50 (0.6%) | 35 (0.4%) | 0.024 | 32 (0.4%) | 34 (0.4%) | 0.004 |
| Preterm or premature birth | 298 (3.3%) | 270 (3.0%) | 0.017 | 247 (3.1%) | 244 (3.0%) | 0.002 |
| Chronic respiratory disease originating in the perinatal period | 146 (1.6%) | 99 (1.1%) | 0.045 | 103 (1.3%) | 98 (1.2%) | 0.006 |
| Heart failure | 94 (1.0%) | 85 (1.0%) | 0.010 | 80 (1.0%) | 82 (1.0%) | 0.002 |
| Antineoplastic use | 451 (5.0%) | 344 (3.8%) | 0.057 | 311 (3.8%) | 319 (3.9%) | 0.005 |

Abbreviations: IQR; interquartile range, SMD; standardized mean difference.

Supplemental Table 5. Characteristics of influenza A(H3N2) confirmed by nucleic acid testing in adults.

|  | Before matching | | | After matching | | |
| --- | --- | --- | --- | --- | --- | --- |
|  | 2025–2026 season (N=7488) | 2024–2025 season (N=6947) | SMD | 2025–2026 season (N=6358) | 2024–2025 season (N=6358) | SMD |
| Age, years |  |  |  |  |  |  |
| Median [IQR] | 59 (43) | 57 (39) | 0.057 | 57 (41) | 58 (40) | 0.010 |
| 18–39 | 2435 (32.5%) | 2257 (32.5%) |  | 2122 (33.4%) | 2111 (33.2%) |  |
| 40–49 | 567 (7.6%) | 646 (9.3%) |  | 534 (8.4%) | 524 (8.2%) |  |
| 50–64 | 1337 (17.9%) | 1478 (21.3%) |  | 1235 (19.4%) | 1234 (19.4%) |  |
| 65–79 | 2098 (28.0%) | 1800 (25.9%) |  | 1694 (26.6%) | 1730 (27.2%) |  |
| ≥80 | 1051 (14.0%) | 766 (11.0%) |  | 773 (12.2%) | 759 (11.9%) |  |
| Sex (Female) | 4278 (57.1%) | 4003 (57.6%) | 0.010 | 3653 (57.5%) | 3657 (57.5%) | 0.001 |
| Race |  |  |  |  |  |  |
| American Indian or Alaska Native | 96 (1.3%) | 49 (0.7%) | 0.058 | 54 (0.8%) | 49 (0.8%) | 0.009 |
| Asian | 184 (2.5%) | 173 (2.5%) | 0.002 | 163 (2.6%) | 160 (2.5%) | 0.003 |
| Black or African American | 1364 (18.2%) | 1286 (18.5%) | 0.008 | 1186 (18.7%) | 1195 (18.8%) | 0.004 |
| Native Hawaiian or Other Pacific Islander | 33 (0.4%) | 31 (0.4%) | 0.001 | 27 (0.4%) | 26 (0.4%) | 0.002 |
| White | 4488 (59.9%) | 4163 (59.9%) | <0.001 | 3865 (60.8%) | 3856 (60.6%) | 0.003 |
| Other Race | 433 (5.8%) | 591 (8.5%) | 0.106 | 424 (6.7%) | 427 (6.7%) | 0.002 |
| Unknown Race | 890 (11.9%) | 654 (9.4%) | 0.080 | 639 (10.1%) | 645 (10.1%) | 0.003 |
| Ethnicity |  |  |  |  |  |  |
| Hispanic or Latino | 1021 (13.6%) | 1163 (16.7%) | 0.087 | 935 (14.7%) | 932 (14.7%) | 0.001 |
| Not Hispanic or Latino | 5888 (78.6%) | 5152 (74.2%) | 0.105 | 4885 (76.8%) | 4893 (77.0%) | 0.003 |
| Unknown Ethnicity | 579 (7.7%) | 632 (9.1%) | 0.049 | 538 (8.5%) | 533 (8.4%) | 0.003 |
| Medical comorbidities |  |  |  |  |  |  |
| High-risk socioeconomic and psychosocial circumstances | 1160 (15.5%) | 885 (12.7%) | 0.079 | 859 (13.5%) | 850 (13.4%) | 0.004 |
| Long term (current) use of steroids | 1087 (14.5%) | 1051 (15.1%) | 0.017 | 921 (14.5%) | 920 (14.5%) | <0.001 |
| Long term (current) use of immunomodulators and immunosuppressants | 442 (5.9%) | 354 (5.1%) | 0.035 | 339 (5.3%) | 337 (5.3%) | 0.001 |
| Long term (current) use of non-steroidal anti-inflammatories | 407 (5.4%) | 399 (5.7%) | 0.013 | 347 (5.5%) | 352 (5.5%) | 0.003 |
| Transplanted organ and tissue status | 272 (3.6%) | 257 (3.7%) | 0.004 | 231 (3.6%) | 231 (3.6%) | <0.001 |
| Chronic lower respiratory diseases | 2888 (38.6%) | 2606 (37.5%) | 0.022 | 2379 (37.4%) | 2382 (37.5%) | 0.001 |
| Tuberculosis | 14 (0.2%) | 18 (0.3%) | 0.015 | 13 (0.2%) | 13 (0.2%) | <0.001 |
| Human immunodeficiency virus infection | 76 (1.0%) | 103 (1.5%) | 0.042 | 74 (1.2%) | 71 (1.1%) | 0.004 |
| Diseases of liver | 1259 (16.8%) | 1052 (15.1%) | 0.046 | 973 (15.3%) | 983 (15.5%) | 0.004 |
| Developmental disorders | 153 (2.0%) | 115 (1.7%) | 0.029 | 110 (1.7%) | 111 (1.7%) | 0.001 |
| Cystic fibrosis | 42 (0.6%) | 50 (0.7%) | 0.020 | 40 (0.6%) | 40 (0.6%) | <0.001 |
| Overweight and obesity | 2436 (32.5%) | 2067 (29.8%) | 0.060 | 1889 (29.7%) | 1921 (30.2%) | 0.011 |
| Diabetes mellitus | 2062 (27.5%) | 1820 (26.2%) | 0.030 | 1661 (26.1%) | 1664 (26.2%) | 0.001 |
| Acute kidney failure and chronic kidney disease | 2184 (29.2%) | 1807 (26.0%) | 0.071 | 1683 (26.5%) | 1708 (26.9%) | 0.009 |
| Cerebral palsy and other paralytic syndromes | 342 (4.6%) | 239 (3.4%) | 0.058 | 251 (3.9%) | 232 (3.6%) | 0.016 |
| Epilepsy and recurrent seizures | 411 (5.5%) | 303 (4.4%) | 0.052 | 283 (4.5%) | 290 (4.6%) | 0.005 |
| Diseases of myoneural junction and muscle | 164 (2.2%) | 117 (1.7%) | 0.037 | 111 (1.7%) | 113 (1.8%) | 0.002 |
| Immunodeficiency and associated syndrome | 703 (9.4%) | 568 (8.2%) | 0.043 | 542 (8.5%) | 542 (8.5%) | <0.001 |
| Sickle-cell disorders | 79 (1.1%) | 63 (0.9%) | 0.015 | 58 (0.9%) | 60 (0.9%) | 0.003 |
| Thalassemia | 37 (0.5%) | 29 (0.4%) | 0.011 | 27 (0.4%) | 29 (0.5%) | 0.005 |
| Heart failure | 1561 (20.8%) | 1286 (18.5%) | 0.059 | 1201 (18.9%) | 1216 (19.1%) | 0.006 |
| Inflammatory polyarthropathies | 1021 (13.6%) | 809 (11.6%) | 0.060 | 776 (12.2%) | 767 (12.1%) | 0.004 |
| Systemic connective tissue disorders | 329 (4.4%) | 222 (3.2%) | 0.063 | 229 (3.6%) | 212 (3.3%) | 0.015 |
| Psoriasis | 160 (2.1%) | 129 (1.9%) | 0.020 | 116 (1.8%) | 124 (2.0%) | 0.009 |
| Multiple sclerosis | 77 (1.0%) | 59 (0.8%) | 0.019 | 59 (0.9%) | 57 (0.9%) | 0.003 |
| Parkinson's disease | 99 (1.3%) | 86 (1.2%) | 0.007 | 82 (1.3%) | 81 (1.3%) | 0.001 |
| Ischemic heart diseases | 2125 (28.4%) | 1726 (24.8%) | 0.080 | 1609 (25.3%) | 1638 (25.8%) | 0.010 |
| Cerebrovascular diseases | 1321 (17.6%) | 935 (13.5%) | 0.116 | 911 (14.3%) | 921 (14.5%) | 0.004 |
| Ulcerative colitis | 113 (1.5%) | 90 (1.3%) | 0.018 | 80 (1.3%) | 86 (1.4%) | 0.008 |
| Crohn's disease | 87 (1.2%) | 77 (1.1%) | 0.005 | 66 (1.0%) | 70 (1.1%) | 0.006 |
| Mood disorders | 2292 (30.6%) | 1965 (28.3%) | 0.051 | 1795 (28.2%) | 1829 (28.8%) | 0.012 |
| Schizophrenia | 245 (3.3%) | 240 (3.5%) | 0.010 | 219 (3.4%) | 213 (3.4%) | 0.005 |
| Nicotine dependence | 1717 (22.9%) | 1654 (23.8%) | 0.021 | 1468 (23.1%) | 1486 (23.4%) | 0.007 |
| Alcohol related disorders | 579 (7.7%) | 524 (7.5%) | 0.007 | 484 (7.6%) | 470 (7.4%) | 0.008 |
| Opioid related disorders | 295 (3.9%) | 318 (4.6%) | 0.032 | 259 (4.1%) | 256 (4.0%) | 0.002 |
| Antineoplastic use | 995 (13.3%) | 765 (11.0%) | 0.070 | 739 (11.6%) | 743 (11.7%) | 0.002 |

Abbreviations: IQR; interquartile range, SMD; standardized mean difference.

Supplemental Table 6. Characteristics of children with influenza A confirmed by nucleic acid testing.

|  | Before matching | | | After matching | | |
| --- | --- | --- | --- | --- | --- | --- |
|  | 2025–2026 season (N=73184) | 2024–2025 season (N=109324) | SMD | 2025–2026 season (N=73147) | 2024–2025 season (N=73147) | SMD |
| Age, years |  |  |  |  |  |  |
| Median [IQR] | 6 (8) | 6 (7) | 0.054 | 6 (8) | 6 (8) | 0.011 |
| <1 | 6051 (8.3%) | 8113 (7.4%) |  | 6051 (8.3%) | 6083 (8.3%) |  |
| 1 year | 6214 (8.5%) | 8951 (8.2%) |  | 6214 (8.5%) | 6255 (8.6%) |  |
| 2–4 | 16133 (22.0%) | 25050 (22.9%) |  | 16133 (22.1%) | 16155 (22.1%) |  |
| 5–11 | 28034 (38.3%) | 46382 (42.4%) |  | 28033 (38.3%) | 27990 (38.3%) |  |
| 12–17 | 16752 (22.9%) | 20828 (19.1%) |  | 16716 (22.9%) | 16664 (22.8%) |  |
| Sex (Female) | 35030 (47.9%) | 52398 (47.9%) | 0.001 | 35014 (47.9%) | 34946 (47.8%) | 0.002 |
| Race |  |  |  |  |  |  |
| American Indian or Alaska Native | 524 (0.7%) | 1150 (1.1%) | 0.036 | 523 (0.7%) | 503 (0.7%) | 0.003 |
| Asian | 3727 (5.1%) | 5209 (4.8%) | 0.015 | 3726 (5.1%) | 3722 (5.1%) | <0.001 |
| Black or African American | 16189 (22.1%) | 24083 (22.0%) | 0.002 | 16183 (22.1%) | 16222 (22.2%) | 0.001 |
| Native Hawaiian or Other Pacific Islander | 1076 (1.5%) | 1590 (1.5%) | 0.001 | 1076 (1.5%) | 1100 (1.5%) | 0.003 |
| White | 39187 (53.5%) | 59942 (54.8%) | 0.026 | 39177 (53.6%) | 39185 (53.6%) | <0.001 |
| Other Race | 6631 (9.1%) | 8704 (8.0%) | 0.039 | 6622 (9.1%) | 6600 (9.0%) | 0.001 |
| Unknown Race | 5850 (8.0%) | 8646 (7.9%) | 0.003 | 5840 (8.0%) | 5815 (7.9%) | 0.001 |
| Ethnicity |  |  |  |  |  |  |
| Hispanic or Latino | 17113 (23.4%) | 22395 (20.5%) | 0.070 | 17083 (23.4%) | 17070 (23.3%) | <0.001 |
| Not Hispanic or Latino | 49017 (67.0%) | 74807 (68.4%) | 0.031 | 49010 (67.0%) | 49141 (67.2%) | 0.004 |
| Unknown Ethnicity | 7054 (9.6%) | 12122 (11.1%) | 0.048 | 7054 (9.6%) | 6936 (9.5%) | 0.005 |
| Medical comorbidities |  |  |  |  |  |  |
| High-risk socioeconomic and psychosocial circumstances | 5342 (7.3%) | 7105 (6.5%) | 0.032 | 5318 (7.3%) | 5169 (7.1%) | 0.008 |
| Long term (current) use of steroids | 1800 (2.5%) | 2370 (2.2%) | 0.019 | 1786 (2.4%) | 1720 (2.4%) | 0.006 |
| Long term (current) use of immunomodulators and immunosuppressants | 440 (0.6%) | 503 (0.5%) | 0.019 | 426 (0.6%) | 405 (0.6%) | 0.004 |
| Long term (current) use of non-steroidal anti-inflammatories | 396 (0.5%) | 588 (0.5%) | <0.001 | 396 (0.5%) | 364 (0.5%) | 0.006 |
| Transplanted organ and tissue status | 158 (0.2%) | 237 (0.2%) | <0.001 | 154 (0.2%) | 138 (0.2%) | 0.005 |
| Chronic lower respiratory diseases | 12356 (16.9%) | 18103 (16.6%) | 0.009 | 12341 (16.9%) | 12378 (16.9%) | 0.001 |
| Tuberculosis | 13 (0.0%) | 23 (0.0%) | 0.002 | 13 (0.0%) | 12 (0.0%) | 0.001 |
| Human immunodeficiency virus infection | 12 (0.0%) | 11 (0.0%) | 0.006 | 10 (0.0%) | 10 (0.0%) | <0.001 |
| Diseases of liver | 472 (0.6%) | 608 (0.6%) | 0.011 | 465 (0.6%) | 430 (0.6%) | 0.006 |
| Pervasive and specific developmental disorders | 11240 (15.4%) | 15911 (14.6%) | 0.023 | 11215 (15.3%) | 11171 (15.3%) | 0.002 |
| Cystic fibrosis | 53 (0.1%) | 92 (0.1%) | 0.004 | 53 (0.1%) | 47 (0.1%) | 0.003 |
| Overweight and obesity | 4292 (5.9%) | 5389 (4.9%) | 0.041 | 4268 (5.8%) | 4268 (5.8%) | <0.001 |
| Diabetes mellitus | 354 (0.5%) | 515 (0.5%) | 0.002 | 353 (0.5%) | 338 (0.5%) | 0.003 |
| Acute kidney failure and chronic kidney disease | 598 (0.8%) | 837 (0.8%) | 0.006 | 591 (0.8%) | 542 (0.7%) | 0.008 |
| Congenital malformations of the circulatory system | 2739 (3.7%) | 3826 (3.5%) | 0.013 | 2730 (3.7%) | 2653 (3.6%) | 0.006 |
| Congenital malformations of the respiratory system | 1139 (1.6%) | 1527 (1.4%) | 0.013 | 1132 (1.5%) | 1079 (1.5%) | 0.006 |
| Congenital malformations of the nervous system | 780 (1.1%) | 1171 (1.1%) | 0.001 | 777 (1.1%) | 695 (1.0%) | 0.011 |
| Congenital malformations of the digestive system | 1601 (2.2%) | 2184 (2.0%) | 0.013 | 1594 (2.2%) | 1594 (2.2%) | <0.001 |
| Cerebral palsy and other paralytic syndromes | 436 (0.6%) | 706 (0.6%) | 0.006 | 435 (0.6%) | 411 (0.6%) | 0.004 |
| Epilepsy and recurrent seizures | 1283 (1.8%) | 1795 (1.6%) | 0.009 | 1281 (1.8%) | 1202 (1.6%) | 0.008 |
| Diseases of myoneural junction and muscle | 146 (0.2%) | 194 (0.2%) | 0.005 | 146 (0.2%) | 119 (0.2%) | 0.009 |
| Immunodeficiency and associated syndromes | 826 (1.1%) | 1150 (1.1%) | 0.007 | 814 (1.1%) | 756 (1.0%) | 0.008 |
| Sickle-cell disorders | 725 (1.0%) | 1058 (1.0%) | 0.002 | 724 (1.0%) | 699 (1.0%) | 0.003 |
| Thalassemia | 218 (0.3%) | 319 (0.3%) | 0.001 | 218 (0.3%) | 202 (0.3%) | 0.004 |
| Preterm or premature birth | 1954 (2.7%) | 2881 (2.6%) | 0.002 | 1951 (2.7%) | 1897 (2.6%) | 0.005 |
| Chronic respiratory disease originating in the perinatal period | 475 (0.6%) | 705 (0.6%) | 0.001 | 473 (0.6%) | 441 (0.6%) | 0.006 |
| Heart failure | 237 (0.3%) | 315 (0.3%) | 0.006 | 233 (0.3%) | 207 (0.3%) | 0.006 |
| Antineoplastic use | 1880 (2.6%) | 2633 (2.4%) | 0.010 | 1870 (2.6%) | 1871 (2.6%) | <0.001 |

Abbreviations: IQR; interquartile range, SMD; standardized mean difference.

Supplemental Table 7. Characteristics of adults with influenza A confirmed by nucleic acid testing.

|  | Before matching | | | After matching | | |
| --- | --- | --- | --- | --- | --- | --- |
|  | 2025–2026 season (N=89101) | 2024–2025 season (N=167690) | SMD | 2025–2026 season (N=89094) | 2024–2025 season (N=89094) | SMD |
| Age, years |  |  |  |  |  |  |
| Median [IQR] | 48 (40) | 50 (33) | 0.047 | 48 (40) | 47 (37) | 0.010 |
| 18–39 | 37173 (41.7%) | 60084 (35.8%) |  | 37168 (41.7%) | 37655 (42.3%) |  |
| 40–49 | 8950 (10.0%) | 23231 (13.9%) |  | 8950 (10.0%) | 8879 (10.0%) |  |
| 50–64 | 15809 (17.7%) | 39042 (23.3%) |  | 15809 (17.7%) | 15791 (17.7%) |  |
| 65–79 | 19532 (21.9%) | 34184 (20.4%) |  | 19532 (21.9%) | 19173 (21.5%) |  |
| ≥80 | 7637 (8.6%) | 11149 (6.6%) |  | 7635 (8.6%) | 7596 (8.5%) |  |
| Sex (Female) | 53031 (59.5%) | 98254 (58.6%) | 0.019 | 53026 (59.5%) | 53012 (59.5%) | <0.001 |
| Race |  |  |  |  |  |  |
| American Indian or Alaska Native | 523 (0.6%) | 1462 (0.9%) | 0.033 | 523 (0.6%) | 506 (0.6%) | 0.003 |
| Asian | 4311 (4.8%) | 6378 (3.8%) | 0.051 | 4308 (4.8%) | 4281 (4.8%) | 0.001 |
| Black or African American | 17427 (19.6%) | 32227 (19.2%) | 0.009 | 17426 (19.6%) | 17478 (19.6%) | 0.001 |
| Native Hawaiian or Other Pacific Islander | 1030 (1.2%) | 1513 (0.9%) | 0.025 | 1029 (1.2%) | 996 (1.1%) | 0.003 |
| White | 57675 (64.7%) | 111221 (66.3%) | 0.034 | 57675 (64.7%) | 57577 (64.6%) | 0.002 |
| Other Race | 4090 (4.6%) | 7829 (4.7%) | 0.004 | 4089 (4.6%) | 4134 (4.6%) | 0.002 |
| Unknown Race | 4045 (4.5%) | 7060 (4.2%) | 0.016 | 4044 (4.5%) | 4122 (4.6%) | 0.004 |
| Ethnicity |  |  |  |  |  |  |
| Hispanic or Latino | 10035 (11.3%) | 16788 (10.0%) | 0.041 | 10031 (11.3%) | 10218 (11.5%) | 0.007 |
| Not Hispanic or Latino | 69587 (78.1%) | 127681 (76.1%) | 0.047 | 69584 (78.1%) | 69415 (77.9%) | 0.005 |
| Unknown Ethnicity | 9479 (10.6%) | 23221 (13.8%) | 0.098 | 9479 (10.6%) | 9461 (10.6%) | 0.001 |
| Medical comorbidities |  |  |  |  |  |  |
| High-risk socioeconomic and psychosocial circumstances | 10516 (11.8%) | 17298 (10.3%) | 0.047 | 10510 (11.8%) | 10184 (11.4%) | 0.011 |
| Long term (current) use of steroids | 7702 (8.6%) | 14364 (8.6%) | 0.003 | 7700 (8.6%) | 7338 (8.2%) | 0.015 |
| Long term (current) use of immunomodulators and immunosuppressants | 2511 (2.8%) | 3963 (2.4%) | 0.029 | 2506 (2.8%) | 2350 (2.6%) | 0.011 |
| Long term (current) use of non-steroidal anti-inflammatories | 3834 (4.3%) | 7542 (4.5%) | 0.009 | 3834 (4.3%) | 3639 (4.1%) | 0.011 |
| Transplanted organ and tissue status | 1316 (1.5%) | 2428 (1.4%) | 0.002 | 1316 (1.5%) | 1244 (1.4%) | 0.007 |
| Chronic lower respiratory diseases | 29028 (32.6%) | 53861 (32.1%) | 0.010 | 29025 (32.6%) | 28681 (32.2%) | 0.008 |
| Tuberculosis | 170 (0.2%) | 345 (0.2%) | 0.003 | 170 (0.2%) | 163 (0.2%) | 0.002 |
| Human immunodeficiency virus infection | 510 (0.6%) | 1001 (0.6%) | 0.003 | 510 (0.6%) | 498 (0.6%) | 0.002 |
| Diseases of liver | 10859 (12.2%) | 19780 (11.8%) | 0.012 | 10855 (12.2%) | 10502 (11.8%) | 0.012 |
| Developmental disorders | 1622 (1.8%) | 2412 (1.4%) | 0.030 | 1620 (1.8%) | 1590 (1.8%) | 0.003 |
| Cystic fibrosis | 135 (0.2%) | 266 (0.2%) | 0.002 | 135 (0.2%) | 125 (0.1%) | 0.003 |
| Overweight and obesity | 26361 (29.6%) | 49708 (29.6%) | 0.001 | 26358 (29.6%) | 26034 (29.2%) | 0.008 |
| Diabetes mellitus | 17128 (19.2%) | 33051 (19.7%) | 0.012 | 17123 (19.2%) | 16668 (18.7%) | 0.013 |
| Acute kidney failure and chronic kidney disease | 13962 (15.7%) | 25260 (15.1%) | 0.017 | 13958 (15.7%) | 13408 (15.0%) | 0.017 |
| Cerebral palsy and other paralytic syndromes | 1917 (2.2%) | 3618 (2.2%) | <0.001 | 1917 (2.2%) | 1785 (2.0%) | 0.010 |
| Epilepsy and recurrent seizures | 2694 (3.0%) | 5187 (3.1%) | 0.004 | 2693 (3.0%) | 2513 (2.8%) | 0.012 |
| Diseases of myoneural junction and muscle | 971 (1.1%) | 1664 (1.0%) | 0.010 | 970 (1.1%) | 940 (1.1%) | 0.003 |
| Immunodeficiency and associated syndrome | 3893 (4.4%) | 6755 (4.0%) | 0.017 | 3888 (4.4%) | 3644 (4.1%) | 0.014 |
| Sickle-cell disorders | 529 (0.6%) | 898 (0.5%) | 0.008 | 528 (0.6%) | 516 (0.6%) | 0.002 |
| Thalassemia | 430 (0.5%) | 616 (0.4%) | 0.018 | 428 (0.5%) | 390 (0.4%) | 0.006 |
| Heart failure | 9158 (10.3%) | 16729 (10.0%) | 0.010 | 9156 (10.3%) | 8705 (9.8%) | 0.017 |
| Inflammatory polyarthropathies | 9431 (10.6%) | 17385 (10.4%) | 0.007 | 9430 (10.6%) | 9075 (10.2%) | 0.013 |
| Systemic connective tissue disorders | 2728 (3.1%) | 4735 (2.8%) | 0.014 | 2726 (3.1%) | 2522 (2.8%) | 0.014 |
| Psoriasis | 1854 (2.1%) | 3556 (2.1%) | 0.003 | 1854 (2.1%) | 1758 (2.0%) | 0.008 |
| Multiple sclerosis | 534 (0.6%) | 1010 (0.6%) | <0.001 | 534 (0.6%) | 494 (0.6%) | 0.006 |
| Parkinson's disease | 688 (0.8%) | 1169 (0.7%) | 0.009 | 688 (0.8%) | 656 (0.7%) | 0.004 |
| Ischemic heart diseases | 15312 (17.2%) | 27873 (16.6%) | 0.015 | 15310 (17.2%) | 14647 (16.4%) | 0.020 |
| Cerebrovascular diseases | 9024 (10.1%) | 15790 (9.4%) | 0.024 | 9020 (10.1%) | 8569 (9.6%) | 0.017 |
| Ulcerative colitis | 980 (1.1%) | 1745 (1.0%) | 0.006 | 980 (1.1%) | 922 (1.0%) | 0.006 |
| Crohn's disease | 777 (0.9%) | 1426 (0.9%) | 0.002 | 777 (0.9%) | 743 (0.8%) | 0.004 |
| Mood disorders | 24174 (27.1%) | 45341 (27.0%) | 0.002 | 24171 (27.1%) | 23801 (26.7%) | 0.009 |
| Schizophrenia | 1975 (2.2%) | 4145 (2.5%) | 0.017 | 1974 (2.2%) | 1864 (2.1%) | 0.009 |
| Nicotine dependence | 17041 (19.1%) | 35022 (20.9%) | 0.044 | 17040 (19.1%) | 16618 (18.7%) | 0.012 |
| Alcohol related disorders | 5236 (5.9%) | 10079 (6.0%) | 0.006 | 5235 (5.9%) | 4942 (5.5%) | 0.014 |
| Opioid related disorders | 2377 (2.7%) | 5104 (3.0%) | 0.023 | 2377 (2.7%) | 2286 (2.6%) | 0.006 |
| Antineoplastic use | 8060 (9.0%) | 13975 (8.3%) | 0.025 | 8055 (9.0%) | 7831 (8.8%) | 0.009 |

Abbreviations: IQR; interquartile range, SMD; standardized mean difference.

Supplemental Table 8. Characteristics of children with all influenza confirmed by nucleic acid testing.

|  | Before matching | | | After matching | | |
| --- | --- | --- | --- | --- | --- | --- |
|  | 2025–2026 season (N=118862) | 2024–2025 season (N=131336) | SMD | 2025–2026 season (N=115187) | 2024–2025 season (N=115187) | SMD |
| Age, years |  |  |  |  |  |  |
| Median [IQR] | 7 (8) | 6 (7) | 0.118 | 7 (8) | 6 (8) | 0.017 |
| <1 | 8004 (6.7%) | 9226 (7.0%) |  | 7991 (6.9%) | 8029 (7.0%) |  |
| 1 year | 8196 (6.9%) | 10049 (7.7%) |  | 8190 (7.1%) | 8188 (7.1%) |  |
| 2–4 | 22161 (18.6%) | 28182 (21.5%) |  | 22133 (19.2%) | 22223 (19.3%) |  |
| 5–11 | 51000 (42.9%) | 57132 (43.5%) |  | 50507 (43.8%) | 50389 (43.7%) |  |
| 12–17 | 29501 (24.8%) | 26747 (20.4%) |  | 26366 (22.9%) | 26358 (22.9%) |  |
| Sex (Female) | 56887 (47.9%) | 62894 (47.9%) | 0.001 | 55093 (47.8%) | 54954 (47.7%) | 0.002 |
| Race |  |  |  |  |  |  |
| American Indian or Alaska Native | 837 (0.7%) | 1276 (1.0%) | 0.029 | 837 (0.7%) | 781 (0.7%) | 0.006 |
| Asian | 5826 (4.9%) | 6491 (4.9%) | 0.002 | 5671 (4.9%) | 5653 (4.9%) | 0.001 |
| Black or African American | 24772 (20.8%) | 28014 (21.3%) | 0.012 | 24156 (21.0%) | 23981 (20.8%) | 0.004 |
| Native Hawaiian or Other Pacific Islander | 1560 (1.3%) | 1722 (1.3%) | <0.001 | 1496 (1.3%) | 1491 (1.3%) | <0.001 |
| White | 64872 (54.6%) | 71353 (54.3%) | 0.005 | 62765 (54.5%) | 62996 (54.7%) | 0.004 |
| Other Race | 11408 (9.6%) | 12007 (9.1%) | 0.016 | 10947 (9.5%) | 10938 (9.5%) | <0.001 |
| Unknown Race | 9587 (8.1%) | 10473 (8.0%) | 0.003 | 9315 (8.1%) | 9347 (8.1%) | 0.001 |
| Ethnicity |  |  |  |  |  |  |
| Hispanic or Latino | 27564 (23.2%) | 27542 (21.0%) | 0.054 | 26023 (22.6%) | 25946 (22.5%) | 0.002 |
| Not Hispanic or Latino | 79937 (67.3%) | 89766 (68.3%) | 0.023 | 77867 (67.6%) | 78096 (67.8%) | 0.004 |
| Unknown Ethnicity | 11361 (9.6%) | 14028 (10.7%) | 0.037 | 11297 (9.8%) | 11145 (9.7%) | 0.004 |
| Medical comorbidities |  |  |  |  |  |  |
| High-risk socioeconomic and psychosocial circumstances | 9199 (7.7%) | 8780 (6.7%) | 0.041 | 8438 (7.3%) | 8339 (7.2%) | 0.003 |
| Long term (current) use of steroids | 3028 (2.5%) | 2798 (2.1%) | 0.028 | 2703 (2.3%) | 2695 (2.3%) | <0.001 |
| Long term use of immunomodulators/ immunosuppressants | 705 (0.6%) | 616 (0.5%) | 0.017 | 607 (0.5%) | 600 (0.5%) | 0.001 |
| Long term (current) use of non-steroidal anti-inflammatories | 631 (0.5%) | 659 (0.5%) | 0.004 | 599 (0.5%) | 599 (0.5%) | <0.001 |
| Transplanted organ and tissue status | 243 (0.2%) | 265 (0.2%) | 0.001 | 225 (0.2%) | 220 (0.2%) | 0.001 |
| Chronic lower respiratory diseases | 21351 (18.0%) | 22149 (16.9%) | 0.029 | 20212 (17.5%) | 20331 (17.7%) | 0.003 |
| Tuberculosis | 24 (0.0%) | 28 (0.0%) | 0.001 | 23 (0.0%) | 21 (0.0%) | 0.001 |
| Human immunodeficiency virus infection | 16 (0.0%) | 12 (0.0%) | 0.004 | 15 (0.0%) | 12 (0.0%) | 0.002 |
| Diseases of liver | 749 (0.6%) | 710 (0.5%) | 0.012 | 677 (0.6%) | 652 (0.6%) | 0.003 |
| Pervasive and specific developmental disorders | 18791 (15.8%) | 19191 (14.6%) | 0.033 | 17814 (15.5%) | 17826 (15.5%) | <0.001 |
| Cystic fibrosis | 99 (0.1%) | 105 (0.1%) | 0.001 | 90 (0.1%) | 95 (0.1%) | 0.002 |
| Overweight and obesity | 7583 (6.4%) | 6711 (5.1%) | 0.055 | 6684 (5.8%) | 6616 (5.7%) | 0.003 |
| Diabetes mellitus | 626 (0.5%) | 614 (0.5%) | 0.008 | 571 (0.5%) | 584 (0.5%) | 0.002 |
| Acute kidney failure and chronic kidney disease | 917 (0.8%) | 924 (0.7%) | 0.008 | 863 (0.7%) | 828 (0.7%) | 0.004 |
| Congenital malformations of the circulatory system | 4302 (3.6%) | 4490 (3.4%) | 0.011 | 4070 (3.5%) | 4087 (3.5%) | 0.001 |
| Congenital malformations of the respiratory system | 1728 (1.5%) | 1761 (1.3%) | 0.010 | 1647 (1.4%) | 1632 (1.4%) | 0.001 |
| Congenital malformations of the nervous system | 1245 (1.0%) | 1345 (1.0%) | 0.002 | 1197 (1.0%) | 1201 (1.0%) | <0.001 |
| Congenital malformations of the digestive system | 2542 (2.1%) | 2563 (2.0%) | 0.013 | 2424 (2.1%) | 2402 (2.1%) | 0.001 |
| Cerebral palsy and other paralytic syndromes | 693 (0.6%) | 807 (0.6%) | 0.004 | 674 (0.6%) | 666 (0.6%) | 0.001 |
| Epilepsy and recurrent seizures | 2085 (1.8%) | 2117 (1.6%) | 0.011 | 1969 (1.7%) | 1924 (1.7%) | 0.003 |
| Diseases of myoneural junction and muscle | 228 (0.2%) | 240 (0.2%) | 0.002 | 218 (0.2%) | 208 (0.2%) | 0.002 |
| Immunodeficiency and associated syndromes | 1327 (1.1%) | 1313 (1.0%) | 0.011 | 1220 (1.1%) | 1180 (1.0%) | 0.003 |
| Sickle-cell disorders | 1166 (1.0%) | 1253 (1.0%) | 0.003 | 1117 (1.0%) | 1078 (0.9%) | 0.003 |
| Thalassemia | 375 (0.3%) | 396 (0.3%) | 0.003 | 355 (0.3%) | 343 (0.3%) | 0.002 |
| Preterm or premature birth | 3089 (2.6%) | 3405 (2.6%) | <0.001 | 2986 (2.6%) | 2938 (2.6%) | 0.003 |
| Chronic respiratory disease originating in the perinatal period | 722 (0.6%) | 818 (0.6%) | 0.002 | 703 (0.6%) | 667 (0.6%) | 0.004 |
| Heart failure | 359 (0.3%) | 338 (0.3%) | 0.008 | 327 (0.3%) | 323 (0.3%) | 0.001 |
| Antineoplastic use | 3263 (2.7%) | 3176 (2.4%) | 0.021 | 2988 (2.6%) | 2915 (2.5%) | 0.004 |

Abbreviations: IQR; interquartile range, SMD; standardized mean difference.

Supplemental Table 9. Characteristics of adults with all influenza confirmed by nucleic acid testing.

|  | Before matching | | | After matching | | |
| --- | --- | --- | --- | --- | --- | --- |
|  | 2025–2026 season (N=112628) | 2024–2025 season (N=185531) | SMD | 2025–2026 season (N=112585) | 2024–2025 season (N=112585) | SMD |
| Age, years |  |  |  |  |  |  |
| Median [IQR] | 43 (37) | 48 (33) | 0.119 | 43 (37) | 43 (36) | 0.016 |
| 18–39 | 51423 (45.7%) | 69024 (37.2%) |  | 51386 (45.6%) | 51731 (45.9%) |  |
| 40–49 | 13126 (11.7%) | 26534 (14.3%) |  | 13125 (11.7%) | 13089 (11.6%) |  |
| 50–64 | 18592 (16.5%) | 41894 (22.6%) |  | 18592 (16.5%) | 18417 (16.4%) |  |
| 65–79 | 21310 (18.9%) | 36251 (19.5%) |  | 21310 (18.9%) | 21155 (18.8%) |  |
| ≥80 | 8177 (7.3%) | 11828 (6.4%) |  | 8172 (7.3%) | 8193 (7.3%) |  |
| Sex (Female) | 67404 (59.8%) | 108980 (58.7%) | 0.023 | 67374 (59.8%) | 67284 (59.8%) | 0.002 |
| Race |  |  |  |  |  |  |
| American Indian or Alaska Native | 683 (0.6%) | 1577 (0.8%) | 0.029 | 683 (0.6%) | 647 (0.6%) | 0.004 |
| Asian | 5348 (4.7%) | 7120 (3.8%) | 0.045 | 5340 (4.7%) | 5331 (4.7%) | <0.001 |
| Black or African American | 21514 (19.1%) | 34681 (18.7%) | 0.010 | 21509 (19.1%) | 21439 (19.0%) | 0.002 |
| Native Hawaiian or Other Pacific Islander | 1350 (1.2%) | 1643 (0.9%) | 0.031 | 1340 (1.2%) | 1308 (1.2%) | 0.003 |
| White | 72756 (64.6%) | 122803 (66.2%) | 0.033 | 72748 (64.6%) | 72755 (64.6%) | <0.001 |
| Other Race | 5617 (5.0%) | 9542 (5.1%) | 0.007 | 5616 (5.0%) | 5682 (5.0%) | 0.003 |
| Unknown Race | 5360 (4.8%) | 8165 (4.4%) | 0.017 | 5349 (4.8%) | 5423 (4.8%) | 0.003 |
| Ethnicity |  |  |  |  |  |  |
| Hispanic or Latino | 13546 (12.0%) | 19600 (10.6%) | 0.046 | 13532 (12.0%) | 13701 (12.2%) | 0.005 |
| Not Hispanic or Latino | 87372 (77.6%) | 140827 (75.9%) | 0.040 | 87344 (77.6%) | 87216 (77.5%) | 0.003 |
| Unknown Ethnicity | 11710 (10.4%) | 25104 (13.5%) | 0.097 | 11709 (10.4%) | 11668 (10.4%) | 0.001 |
| Medical comorbidities |  |  |  |  |  |  |
| High-risk socioeconomic and psychosocial circumstances | 13545 (12.0%) | 19122 (10.3%) | 0.055 | 13509 (12.0%) | 12984 (11.5%) | 0.014 |
| Long term (current) use of steroids | 9368 (8.3%) | 15638 (8.4%) | 0.004 | 9355 (8.3%) | 9026 (8.0%) | 0.011 |
| Long term (current) use of immunomodulators and immunosuppressants | 3016 (2.7%) | 4248 (2.3%) | 0.025 | 3002 (2.7%) | 2801 (2.5%) | 0.011 |
| Long term (current) use of non-steroidal anti-inflammatories | 4827 (4.3%) | 8196 (4.4%) | 0.006 | 4827 (4.3%) | 4586 (4.1%) | 0.011 |
| Transplanted organ and tissue status | 1563 (1.4%) | 2588 (1.4%) | 0.001 | 1559 (1.4%) | 1497 (1.3%) | 0.005 |
| Chronic lower respiratory diseases | 35785 (31.8%) | 58826 (31.7%) | 0.001 | 35766 (31.8%) | 35462 (31.5%) | 0.006 |
| Tuberculosis | 224 (0.2%) | 373 (0.2%) | <0.001 | 224 (0.2%) | 199 (0.2%) | 0.005 |
| Human immunodeficiency virus infection | 644 (0.6%) | 1080 (0.6%) | 0.001 | 644 (0.6%) | 597 (0.5%) | 0.006 |
| Diseases of liver | 13285 (11.8%) | 21659 (11.7%) | 0.004 | 13269 (11.8%) | 12765 (11.3%) | 0.014 |
| Developmental disorders | 2148 (1.9%) | 2705 (1.5%) | 0.035 | 2134 (1.9%) | 2039 (1.8%) | 0.006 |
| Cystic fibrosis | 179 (0.2%) | 280 (0.2%) | 0.002 | 179 (0.2%) | 171 (0.2%) | 0.002 |
| Overweight and obesity | 32917 (29.2%) | 54508 (29.4%) | 0.003 | 32899 (29.2%) | 32610 (29.0%) | 0.006 |
| Diabetes mellitus | 20012 (17.8%) | 35488 (19.1%) | 0.035 | 20006 (17.8%) | 19604 (17.4%) | 0.009 |
| Acute kidney failure and chronic kidney disease | 16005 (14.2%) | 27068 (14.6%) | 0.011 | 15996 (14.2%) | 15438 (13.7%) | 0.014 |
| Cerebral palsy and other paralytic syndromes | 2231 (2.0%) | 3890 (2.1%) | 0.008 | 2231 (2.0%) | 2095 (1.9%) | 0.009 |
| Epilepsy and recurrent seizures | 3346 (3.0%) | 5657 (3.0%) | 0.005 | 3344 (3.0%) | 3171 (2.8%) | 0.009 |
| Diseases of myoneural junction and muscle | 1159 (1.0%) | 1807 (1.0%) | 0.006 | 1157 (1.0%) | 1105 (1.0%) | 0.005 |
| Immunodeficiency and associated syndrome | 4643 (4.1%) | 7287 (3.9%) | 0.010 | 4630 (4.1%) | 4364 (3.9%) | 0.012 |
| Sickle-cell disorders | 700 (0.6%) | 977 (0.5%) | 0.013 | 696 (0.6%) | 676 (0.6%) | 0.002 |
| Thalassemia | 545 (0.5%) | 690 (0.4%) | 0.017 | 538 (0.5%) | 493 (0.4%) | 0.006 |
| Heart failure | 10312 (9.2%) | 17805 (9.6%) | 0.015 | 10309 (9.2%) | 9938 (8.8%) | 0.012 |
| Inflammatory polyarthropathies | 10868 (9.6%) | 18647 (10.1%) | 0.013 | 10860 (9.6%) | 10578 (9.4%) | 0.009 |
| Systemic connective tissue disorders | 3227 (2.9%) | 5122 (2.8%) | 0.006 | 3222 (2.9%) | 3085 (2.7%) | 0.007 |
| Psoriasis | 2252 (2.0%) | 3901 (2.1%) | 0.007 | 2251 (2.0%) | 2175 (1.9%) | 0.005 |
| Multiple sclerosis | 639 (0.6%) | 1110 (0.6%) | 0.004 | 639 (0.6%) | 613 (0.5%) | 0.003 |
| Parkinson's disease | 749 (0.7%) | 1247 (0.7%) | 0.001 | 749 (0.7%) | 696 (0.6%) | 0.006 |
| Ischemic heart diseases | 17264 (15.3%) | 29678 (16.0%) | 0.018 | 17258 (15.3%) | 16764 (14.9%) | 0.012 |
| Cerebrovascular diseases | 10206 (9.1%) | 16980 (9.2%) | 0.003 | 10198 (9.1%) | 9746 (8.7%) | 0.014 |
| Ulcerative colitis | 1186 (1.1%) | 1908 (1.0%) | 0.002 | 1182 (1.0%) | 1138 (1.0%) | 0.004 |
| Crohn's disease | 959 (0.9%) | 1564 (0.8%) | 0.001 | 958 (0.9%) | 940 (0.8%) | 0.002 |
| Mood disorders | 30752 (27.3%) | 50050 (27.0%) | 0.007 | 30724 (27.3%) | 30353 (27.0%) | 0.007 |
| Schizophrenia | 2469 (2.2%) | 4509 (2.4%) | 0.016 | 2469 (2.2%) | 2323 (2.1%) | 0.009 |
| Nicotine dependence | 21785 (19.3%) | 38151 (20.6%) | 0.031 | 21780 (19.3%) | 21413 (19.0%) | 0.008 |
| Alcohol related disorders | 6592 (5.9%) | 11053 (6.0%) | 0.004 | 6588 (5.9%) | 6251 (5.6%) | 0.013 |
| Opioid related disorders | 3047 (2.7%) | 5586 (3.0%) | 0.018 | 3047 (2.7%) | 2877 (2.6%) | 0.009 |
| Antineoplastic use | 9852 (8.7%) | 15002 (8.1%) | 0.024 | 9832 (8.7%) | 9480 (8.4%) | 0.011 |

Abbreviations: IQR; interquartile range, SMD; standardized mean difference.

Supplemental Table 10. Exploratory analyses: Clinical outcomes of all influenza A.

|  | Pediatric cohort | | | | |
| --- | --- | --- | --- | --- | --- |
|  | 2025–26 season (N=73147) | 2024–25 season (N=73147) | OR [95%CI] | RD/1,000 cases [95%CI] | p |
| Death | 17 (0.02%) | 19 (0.03%) | 0.90 [0.47–1.72] | -0.03 [-0.14–0.19] | 0.739 |
| Critical care admission | 664 (0.91%) | 563 (0.77%) | 1.18 [1.06–1.32] | 1.38 [0.42–2.46] | 0.004 |
| Hospitalization | 3362 (4.60%) | 3599 (4.92%) | 0.93 [0.89–0.98] | -3.25 [-5.31–-1.08] | 0.004 |
| Mechanical ventilation | 437 (0.60%) | 493 (0.67%) | 0.89 [0.78–1.01] | -0.77 [-1.48–0.05] | 0.065 |
| Vasopressor | 986 (1.35%) | 1060 (1.45%) | 0.93 [0.85–1.01] | -1.01 [-2.13–0.20] | 0.099 |
|  | Adult cohort | | | | |
|  | 2025–26 season (N=89094) | 2024–25 season (N=89094) | OR [95%CI] | RD/1,000 cases [95%CI] | p |
| Death | 983 (1.10%) | 1073 (1.20%) | 0.92 [0.84–1.00] | -1.01 [-1.91–-0.02] | 0.046 |
| Critical care admission | 4264 (4.79%) | 4107 (4.61%) | 1.04 [1.00–1.09] | 1.75 [-0.18–3.83] | 0.079 |
| Hospitalization | 20077 (22.53%) | 19371 (21.74%) | 1.05 [1.02–1.07] | 7.83 [4.13–11.96] | <0.001 |
| Mechanical ventilation | 3549 (3.98%) | 3603 (4.04%) | 0.98 [0.94–1.03] | -0.61 [-2.39–1.25] | 0.515 |
| Vasopressor | 4335 (4.87%) | 4573 (5.13%) | 0.95 [0.91–0.99] | -2.67 [-4.62–-0.67] | 0.010 |

Abbreviations: CI; confidence interval, NA; not assessed, OR; odds ratio; RD, risk difference.

Supplemental Table 11. Exploratory analyses: Clinical outcomes of all influenza.

|  | Pediatric cohort | | | | |
| --- | --- | --- | --- | --- | --- |
|  | 2025–26 season (N=115187) | 2024–25 season (N=115187) | OR [95%CI] | RD/1,000 cases [95%CI] | p |
| Death | 24 (0.02%) | 22 (0.02%) | 1.09 [0.61–1.95] | 0.02 [-0.07–0.18] | 0.768 |
| Critical care admission | 965 (0.84%) | 868 (0.75%) | 1.11 [1.02–1.22] | 0.84 [0.11–1.64] | 0.023 |
| Hospitalization | 5237 (4.55%) | 5459 (4.74%) | 0.96 [0.92–1.00] | -1.94 [-3.60–-0.19] | 0.028 |
| Mechanical ventilation | 616 (0.53%) | 738 (0.64%) | 0.83 [0.75–0.93] | -1.06 [-1.60–-0.45] | 0.001 |
| Vasopressor | 1598 (1.39%) | 1602 (1.39%) | 1.00 [0.93–1.07] | -0.03 [-0.96–0.96] | 0.943 |
|  | Adult cohort | | | | |
|  | 2025–26 season (N=112585) | 2024–25 season (N=112585) | OR [95%CI] | RD/1,000 cases [95%CI] | p |
| Death | 1083 (0.96%) | 1214 (1.08%) | 0.89 [0.82–0.97] | -1.16 [-1.92–-0.35] | 0.006 |
| Critical care admission | 5027 (4.47%) | 4938 (4.39%) | 1.02 [0.98–1.06] | 0.79 [-0.88–2.54] | 0.362 |
| Hospitalization | 23505 (20.88%) | 22987 (20.42%) | 1.03 [1.01–1.05] | 4.70 [1.23–7.96] | 0.007 |
| Mechanical ventilation | 3975 (3.53%) | 4121 (3.66%) | 0.96 [0.92–1.01] | -1.28 [-2.78–0.26] | 0.098 |
| Vasopressor | 5167 (4.59%) | 5419 (4.81%) | 0.95 [0.92–0.99] | -2.26 [-3.90–-0.48] | 0.012 |

Abbreviations: CI; confidence interval, NA; not assessed, OR; odds ratio; RD, risk difference.

Supplemental Table 12. Exploratory analyses: Clinical outcomes of influenza A(H3N2) among those with documented vaccination between 8 months and 14 days before infection.

|  | Pediatric cohort | | | | |
| --- | --- | --- | --- | --- | --- |
|  | 2025–26 season (N=361) | 2024–25 season (N=361) | OR [95%CI] | RD/1,000 cases [95%CI] | p |
| Death | NA | NA | NA | NA | NA |
| Critical care admission | NA | NA | NA | NA | NA |
| Hospitalization | 91 (25.21%) | 73 (20.22%) | 1.33 [0.94–1.89] | 49.95 [-10.11–128.41] | 0.110 |
| Mechanical ventilation | 12 (3.32%) | 16 (4.43%) | 0.74 [0.35–1.59] | -11.08 [-28.37–24.95] | 0.441 |
| Vasopressor | 21 (5.82%) | 25 (6.93%) | 0.83 [0.46–1.51] | -11.08 [-36.08–32.76] | 0.542 |
|  | Adult cohort | | | | |
|  | 2025–26 season (N=180) | 2024–25 season (N=180) | OR [95%CI] | RD/1,000 cases [95%CI] | p |
| Death | NA | NA | NA | NA | NA |
| Critical care admission | 33 (18.33%) | 37 (20.56%) | 0.87 [0.51–1.46] | -22.20 [-85.31–73.79] | 0.594 |
| Hospitalization | 114 (63.33%) | 109 (60.56%) | 1.13 [0.74–1.72] | 27.86 [-67.22–139.28] | 0.587 |
| Mechanical ventilation | 23 (12.78%) | 38 (21.11%) | 0.55 [0.31–0.96] | -83.39 [-131.73–-5.70] | 0.035 |
| Vasopressor | 44 (24.44%) | 40 (22.22%) | 1.13 [0.69–1.85] | 22.22 [-54.22–133.56] | 0.618 |

Abbreviations: CI; confidence interval, NA; not assessed, OR; odds ratio; RD, risk difference.

Supplemental Table 13. Exploratory analyses: Clinical outcomes of influenza A(H3N2) among those without documented vaccination between 8 months and 14 days before infection.

|  | Pediatric cohort | | | | |
| --- | --- | --- | --- | --- | --- |
|  | 2025–26 season (N=7589) | 2024–25 season (N=7589) | OR [95%CI] | RD/1,000 cases [95%CI] | p |
| Death | NA | NA | NA | NA | NA |
| Critical care admission | 189 (2.49%) | 252 (3.32%) | 0.74 [0.61–0.90] | -8.30 [-12.52–-3.22] | 0.002 |
| Hospitalization | 1022 (13.47%) | 1079 (14.22%) | 0.94 [0.86–1.03] | -7.54 [-17.77–3.55] | 0.180 |
| Mechanical ventilation | 215 (2.83%) | 204 (2.69%) | 1.06 [0.87–1.28] | 1.45 [-3.41–7.34] | 0.586 |
| Vasopressor | 189 (2.49%) | 217 (2.86%) | 0.87 [0.71–1.06] | -3.69 [-8.03–1.60] | 0.159 |
|  | Adult cohort | | | | |
|  | 2025–26 season (N=6131) | 2024–25 season (N=6131) | OR [95%CI] | RD/1,000 cases [95%CI] | p |
| Death | 193 (3.15%) | 214 (3.49%) | 0.90 [0.74–1.10] | -3.42 [-8.90–3.21] | 0.290 |
| Critical care admission | 743 (12.12%) | 803 (13.10%) | 0.92 [0.82–1.02] | -9.82 [-20.56–2.10] | 0.103 |
| Hospitalization | 2830 (46.16%) | 2833 (46.21%) | 1.00 [0.93–1.07] | -0.46 [-18.02–17.56] | 0.957 |
| Mechanical ventilation | 680 (11.09%) | 704 (11.48%) | 0.96 [0.86–1.08] | -3.90 [-14.35–7.69] | 0.493 |
| Vasopressor | 682 (11.12%) | 727 (11.86%) | 0.93 [0.83–1.04] | -7.35 [-17.79–4.15] | 0.203 |

Abbreviations: CI; confidence interval, NA; not assessed, OR; odds ratio; RD, risk difference.

Supplemental Table 14. Exploratory analyses: Clinical outcomes of influenza A(H3N2) among those with documented receipt of anti-influenza medication within 7 days before infection.

|  | Pediatric cohort | | | | |
| --- | --- | --- | --- | --- | --- |
|  | 2025–26 season (N=2274) | 2024–25 season (N=2274) | OR [95%CI] | RD/1,000 cases [95%CI] | p |
| Death | NA | NA | NA | NA | NA |
| Critical care admission | 107 (4.71%) | 99 (4.35%) | 1.09 [0.82–1.44] | 3.53 [-7.53–17.94] | 0.568 |
| Hospitalization | 463 (20.36%) | 438 (19.26%) | 1.07 [0.93–1.24] | 10.98 [-11.56–36.21] | 0.352 |
| Mechanical ventilation | 113 (4.97%) | 132 (5.80%) | 0.85 [0.66–1.10] | -8.36 [-19.16–5.40] | 0.212 |
| Vasopressor | 107 (4.71%) | 97 (4.27%) | 1.11 [0.84–1.47] | 4.39 [-6.70–18.90] | 0.474 |
|  | Adult cohort | | | | |
|  | 2025–26 season (N=2749) | 2024–25 season (N=2749) | OR [95%CI] | RD/1,000 cases [95%CI] | p |
| Death | 112 (4.07%) | 133 (4.84%) | 0.84 [0.65–1.08] | -7.64 [-16.50–3.73] | 0.170 |
| Critical care admission | 442 (16.08%) | 512 (18.62%) | 0.84 [0.73–0.96] | -25.52 [-43.02–-5.77] | 0.013 |
| Hospitalization | 1639 (59.62%) | 1643 (59.77%) | 0.99 [0.89–1.11] | -1.20 [-26.90–25.10] | 0.912 |
| Mechanical ventilation | 392 (14.26%) | 458 (16.66%) | 0.83 [0.72–0.96] | -23.99 [-40.65–-5.16] | 0.014 |
| Vasopressor | 392 (14.26%) | 451 (16.41%) | 0.85 [0.73–0.98] | -21.49 [-38.23–-2.46] | 0.027 |

Abbreviations: CI; confidence interval, NA; not assessed, OR; odds ratio; RD, risk difference.

Supplemental Table 15. Exploratory analyses: Clinical outcomes of influenza A(H3N2) among those without documented receipt of anti-influenza medication within 7 days before infection.

|  | Pediatric cohort | | | | |
| --- | --- | --- | --- | --- | --- |
|  | 2025–26 season (N=5513) | 2024–25 season (N=5513) | OR [95%CI] | RD/1,000 cases [95%CI] | p |
| Death | NA | NA | NA | NA | NA |
| Critical care admission | 138 (2.50%) | 95 (1.72%) | 1.46 [1.13–1.91] | 7.81 [2.10–15.18] | 0.004 |
| Hospitalization | 686 (12.44%) | 645 (11.70%) | 1.07 [0.96–1.20] | 7.49 [-4.45–20.59] | 0.231 |
| Mechanical ventilation | 95 (1.72%) | 97 (1.76%) | 0.98 [0.74–1.30] | -0.37 [-4.57–5.21] | 0.884 |
| Vasopressor | 130 (2.36%) | 116 (2.10%) | 1.12 [0.87–1.45] | 2.55 [-2.63–9.15] | 0.367 |
|  | Adult cohort | | | | |
|  | 2025–26 season (N=3392) | 2024–25 season (N=3392) | OR [95%CI] | RD/1,000 cases [95%CI] | p |
| Death | 72 (2.12%) | 85 (2.51%) | 0.84 [0.61–1.16] | -3.83 [-9.50–3.88] | 0.294 |
| Critical care admission | 295 (8.70%) | 293 (8.64%) | 1.01 [0.85–1.19] | 0.60 [-11.83–15.12] | 0.931 |
| Hospitalization | 1235 (36.41%) | 1192 (35.14%) | 1.06 [0.96–1.17] | 12.65 [-9.84–36.55] | 0.276 |
| Mechanical ventilation | 257 (7.58%) | 245 (7.22%) | 1.05 [0.88–1.26] | 3.54 [-8.23–17.41] | 0.578 |
| Vasopressor | 307 (9.05%) | 288 (8.49%) | 1.07 [0.91–1.27] | 5.60 [-7.30–20.63] | 0.415 |

Abbreviations: CI; confidence interval, NA; not assessed, OR; odds ratio; RD, risk difference.

Supplemental Table 16. Exploratory analyses: Clinical outcomes of influenza A(H3N2) in academic healthcare organizations.

|  | Pediatric cohort | | | | |
| --- | --- | --- | --- | --- | --- |
|  | 2025–26 season (N=5593) | 2024–25 season (N=5593) | OR [95%CI] | RD/1,000 cases [95%CI] | p |
| Death | NA | NA | NA | NA | NA |
| Critical care admission | 144 (2.57%) | 110 (1.97%) | 1.32 [1.03–1.69] | 6.08 [0.47–13.24] | 0.031 |
| Hospitalization | 766 (13.70%) | 844 (15.09%) | 0.89 [0.80–0.99] | -13.88 [-25.80–-0.91] | 0.036 |
| Mechanical ventilation | 148 (2.65%) | 179 (3.20%) | 0.82 [0.66–1.03] | -5.54 [-10.66–0.80] | 0.082 |
| Vasopressor | 149 (2.66%) | 144 (2.57%) | 1.04 [0.82–1.31] | 0.90 [-4.51–7.65] | 0.767 |
|  | Adult cohort | | | | |
|  | 2025–26 season (N=4765) | 2024–25 season (N=4765) | OR [95%CI] | RD/1,000 cases [95%CI] | p |
| Death | 131 (2.75%) | 157 (3.29%) | 0.83 [0.66–1.05] | -5.47 [-11.07–1.61] | 0.120 |
| Critical care admission | 528 (11.08%) | 531 (11.14%) | 0.99 [0.87–1.13] | -0.67 [-12.48–12.70] | 0.927 |
| Hospitalization | 2020 (42.39%) | 1955 (41.03%) | 1.06 [0.98–1.15] | 13.54 [-6.15–34.05] | 0.177 |
| Mechanical ventilation | 455 (9.55%) | 482 (10.12%) | 0.94 [0.82–1.07] | -5.66 [-16.59–6.68] | 0.353 |
| Vasopressor | 499 (10.47%) | 546 (11.46%) | 0.90 [0.80–1.03] | -9.85 [-21.20–2.86] | 0.123 |

Abbreviations: CI; confidence interval, NA; not assessed, OR; odds ratio; RD, risk difference.

Supplemental Table 17. Exploratory analyses: Clinical outcomes of influenza A(H3N2) in non-academic healthcare organizations.

|  | Pediatric cohort | | | | |
| --- | --- | --- | --- | --- | --- |
|  | 2025–26 season (N=2210) | 2024–25 season (N=2210) | OR [95%CI] | RD/1,000 cases [95%CI] | p |
| Death | NA | NA | NA | NA | NA |
| Critical care admission | 115 (5.20%) | 77 (3.48%) | 1.52 [1.13–2.04] | 17.21 [4.39–34.18] | 0.005 |
| Hospitalization | 383 (17.33%) | 254 (11.49%) | 1.61 [1.36–1.92] | 58.39 [34.59–85.85] | <0.001 |
| Mechanical ventilation | 61 (2.76%) | 53 (2.40%) | 1.16 [0.80–1.68] | 3.62 [-4.80–15.71] | 0.448 |
| Vasopressor | 83 (3.76%) | 68 (3.08%) | 1.23 [0.89–1.70] | 6.80 [-3.35–20.71] | 0.214 |
|  | Adult cohort | | | | |
|  | 2025–26 season (N=1367) | 2024–25 season (N=1367) | OR [95%CI] | RD/1,000 cases [95%CI] | p |
| Death | 61 (4.46%) | 63 (4.61%) | 0.97 [0.67–1.39] | -1.47 [-14.47–16.87] | 0.854 |
| Critical care admission | 217 (15.87%) | 257 (18.80%) | 0.82 [0.67–0.99] | -29.33 [-53.39–-0.94] | 0.043 |
| Hospitalization | 868 (63.50%) | 859 (62.84%) | 1.03 [0.88–1.20] | 6.28 [-28.91–43.99] | 0.721 |
| Mechanical ventilation | 219 (16.02%) | 221 (16.17%) | 0.99 [0.81–1.21] | -1.46 [-26.68–28.45] | 0.917 |
| Vasopressor | 186 (13.61%) | 175 (12.80%) | 1.07 [0.86–1.34] | 8.07 [-15.75–36.87] | 0.534 |

Abbreviations: CI; confidence interval, NA; not assessed, OR; odds ratio; RD, risk difference.

Included healthcare organizations with unknown academic status.

Supplemental Table 18. Exploratory analyses: Clinical outcomes of influenza A(H3N2) by excluding outcomes which occurred at the same day to infection detection.

|  | Pediatric cohort | | | | |
| --- | --- | --- | --- | --- | --- |
|  | 2025–26 season (N=7402) | 2024–25 season (N=7402) | OR [95%CI] | RD/1,000 cases [95%CI] | p |
| Death | NA | NA | NA | NA | NA |
| Critical care admission | 143 (1.93%) | 112 (1.51%) | 1.28 [1.00–1.65] | 4.19 [-0.02–9.56] | 0.050 |
| Hospitalization | 883 (11.93%) | 754 (10.19%) | 1.19 [1.08–1.32] | 17.42 [6.93–28.93] | 0.001 |
| Mechanical ventilation | 103 (1.39%) | 118 (1.59%) | 0.87 [0.67–1.14] | -2.02 [-5.24–2.15] | 0.309 |
| Vasopressor | 174 (2.35%) | 154 (2.08%) | 1.13 [0.91–1.41] | 2.70 [-1.83–8.32] | 0.264 |
|  | Adult cohort | | | | |
|  | 2025–26 season (N=6358) | 2024–25 season (N=6358) | OR [95%CI] | RD/1,000 cases [95%CI] | p |
| Death | 183 (2.88%) | 216 (3.40%) | 0.84 [0.69–1.03] | -5.20 [-10.26–0.95] | 0.093 |
| Critical care admission | 431 (6.78%) | 467 (7.35%) | 0.92 [0.80–1.05] | -5.66 [-13.74–3.45] | 0.213 |
| Hospitalization | 2535 (39.87%) | 2498 (39.29%) | 1.03 [0.95–1.10] | 5.89 [-11.00–23.18] | 0.502 |
| Mechanical ventilation | 458 (7.20%) | 518 (8.15%) | 0.88 [0.77–1.00] | -9.45 [-17.60–-0.16] | 0.046 |
| Vasopressor | 575 (9.04%) | 642 (10.10%) | 0.89 [0.79–1.00] | -10.50 [-19.69–-0.30] | 0.043 |

Abbreviations: CI; confidence interval, NA; not assessed, OR; odds ratio; RD, risk difference.

Supplemental Table 19. Distribution of timing between vaccination and infection among vaccinated participants (prior to matching).

|  | 2025–26 season | | 2024–25 season | |
| --- | --- | --- | --- | --- |
|  | Children | Adults | Children | Adults |
| 14 days–2 months | 208 | 99 | 116 | 41 |
| 2–4 months | 276 | 168 | 263 | 128 |
| 4–6 months | 59 | 32 | 113 | 57 |
| 6–8 months | 13 | ≤10 | ≤10 | ≤10 |

Presented as the number of participants.

≤10 events precluded total number and percent estimations.
